# Network Pharmacology-Guided Phytochemical Polypharmacology: Integrating Molecular Mechanisms, Experimental Validation and Clinical Translation

**DOI:** 10.3390/ijms27198683

**Published:** 2026-09-28

**Authors:** Saahil A. Singh, Prabodh Wankhade, Rajesh Godvarthi, Rupesh Warbhe, Prashanth Khartade, Mohd Basheeruddin, Shilpa Narwade

**Affiliations:** 1 Department of Pharmacology, Jawaharlal Nehru Medical College, Adani Institute of Higher Education and Research, Wardha 442107, Maharashtra, India; saahilsingh5559@gmail.com (S.A.S.);; 2 Department of Paediatrics, Jawaharlal Nehru Medical College, Adani Institute of Higher Education and Research, Wardha 442107, Maharashtra, India; 3 Department of Biochemistry, Jawaharlal Nehru Medical College, Adani Institute of Higher Education and Research, Wardha 442107, Maharashtra, India; 4 Department of Anatomy, Jawaharlal Nehru Medical College, Adani Institute of Higher Education and Research, Wardha 442107, Maharashtra, India

**Keywords:** network pharmacology, phytochemical polypharmacology, target engagement, pharmacokinetics, pharmacodynamics, translational pharmacology, curcumin, berberine, EGCG, resveratrol

## Abstract

Network pharmacology offers a systems-level framework for prioritising potential multi-target actions of phytochemicals, but predicted network convergence alone does not establish pharmacologically relevant polypharmacology. This structured narrative review evaluates curcumin, berberine, epigallocatechin-3-gallate (EGCG), and resveratrol as representative case studies, drawing on substantial mechanistic, pharmacokinetic, and translational literature. Evidence is appraised across computational prediction, biochemical interaction, cellular pathway modulation, target engagement or functional validation, in vivo pharmacology, and human evidence, with an emphasis on exposure compatibility. Across the four compounds, overlapping effects on inflammatory, metabolic, stress-response, and growth-regulatory pathways are supported predominantly by cellular and pathway-level studies. In contrast, direct target engagement, causal contribution, and clinically achievable exposure are less consistently demonstrated. Human pharmacokinetic and tissue-exposure studies show that systemic parent-compound concentrations may differ markedly from those used in mechanistic experiments, although local exposure and metabolites can modify this relationship. Clinical studies support selected pharmacodynamic or metabolic effects but do not establish that therapeutic benefit results from the simultaneous engagement of the broad, predicted target networks. Emerging computational, experimental, and delivery approaches can strengthen hypothesis prioritisation but do not replace mechanistic validation. We propose an exposure-aware, evidence-tiered framework in which clinically meaningful phytochemical polypharmacology requires convergence of achievable exposure, target engagement, causal pharmacodynamics, and clinical outcomes.

## 1. Introduction

Historically, pharmaceutical drug discovery has been strongly influenced by the single-target paradigm, in which therapeutic agents are designed to modulate defined molecular targets associated with disease [1,2]. Although this approach has been highly productive, its application to complex multifactorial disorders can be challenging because disease phenotypes frequently arise from interactions among multiple signalling pathways, regulatory feedback loops, compensatory mechanisms, and interconnected biological networks [1,3,4]. Disorders such as cancer, Alzheimer’s disease (AD), type 2 diabetes mellitus (T2DM), and chronic inflammatory diseases therefore provide a rationale for pharmacological approaches that consider molecular systems rather than isolated targets alone [1,3].

Network pharmacology has emerged as a systems-level framework for investigating the molecular basis of multi-component therapeutics, including natural products and phytochemicals [1,5,6]. It integrates compound–target associations, protein–protein interaction (PPI) networks, pathway enrichment, and disease-associated molecular modules to prioritise relationships between chemical perturbations and biological networks [3,5,6]. Such analyses frequently identify signalling pathways including NF-κB, PI3K/Akt, MAPK, Nrf2, and AMPK/mTOR [5,7]. However, pathway enrichment, network centrality, docking predictions, and computationally inferred compound–target associations do not establish simultaneous target engagement, causal pathway involvement, or therapeutic efficacy [1,5,6]. Network-pharmacology outputs should therefore be regarded primarily as hypothesis-generating evidence requiring experimental and pharmacological validation [1,5].

This distinction is particularly important in the context of polypharmacology. In this review, polypharmacology refers to the functionally relevant engagement of multiple molecular targets by a therapeutic agent, resulting in modulation of disease-associated biological processes [2,8,9]. It differs conceptually from combination therapy, in which multiple molecular entities are administered concurrently, and from nonspecific molecular promiscuity, in which broad molecular interactions occur without evidence that they contribute meaningfully to the therapeutic phenotype [2,8,10]. Accordingly, the presence of multiple predicted targets is not sufficient to establish therapeutically useful polypharmacology. For this review, the credibility of a proposed multi-target mechanism is evaluated in relation to interaction strength and selectivity, pharmacologically relevant exposure, target occupancy or binding kinetics where measurable, tissue accessibility, active metabolites, and evidence linking target modulation to the biological phenotype [1,8,9]. Phytochemicals provide an informative setting in which to examine these issues because many plant-derived compounds affect multiple molecular and cellular processes [7,11]. Nevertheless, describing phytochemicals as inherently polypharmacological agents may overstate the available evidence. Reported associations with nuclear receptors, protein kinases, redox-regulated proteins, transcriptional regulators, and epigenetic enzymes may reflect direct molecular interaction, chemical reactivity, indirect signalling effects, altered protein expression, metabolite activity, or experimental artefacts [5,11,12]. Thus, broad target profiles should not automatically be interpreted as coordinated therapeutic polypharmacology. Instead, individual compound–target relationships should be assessed according to the strength and type of supporting evidence.

Curcumin, berberine, epigallocatechin-3-gallate (EGCG), and resveratrol are examined here as representative case studies rather than an exhaustive catalogue of phytochemical polypharmacology. These compounds were selected because they have generated extensive literature spanning biochemical and cellular pharmacology, pharmacokinetics, animal studies, formulation research, and clinical investigation [13,14,15,16]. Their recurring associations with inflammatory, metabolic, oxidative-stress, and growth-regulatory pathways provide an opportunity to examine whether apparent network convergence reflects validated multi-target pharmacology, downstream convergence from a smaller number of primary molecular events, context-dependent signalling responses, or limitations of computational and pathway-based analyses [7].

These interpretive issues are particularly relevant to frequently reported signalling pathways, including NF-κB, PI3K/Akt, MAPK, Nrf2, and AMPK/mTOR [7]. Proposed molecular mechanisms involving targets such as SIRT1, IKKβ, and DNMT1 require additional caution because the strength of evidence for direct engagement differs substantially among compounds and experimental systems [12,17,18]. Pathway-level convergence may indicate biologically meaningful activity, but it does not establish that multiple upstream targets are simultaneously engaged or that each pathway contributes causally to the observed phenotype [5]. Direct biochemical interactions, downstream cellular responses, and computationally inferred associations should therefore be interpreted as distinct levels of mechanistic evidence [5,19].

A second major translational consideration is the exposure–activity relationship. For several of the phytochemicals considered here, biological effects observed in cellular systems occur at concentrations that may exceed those achieved as free parent compound in human systemic circulation. Limited oral bioavailability, extensive metabolism, protein binding, tissue distribution, and formation of metabolites can substantially alter the relationship between administered dose, systemic exposure, target engagement, and pharmacodynamic response [14,20,21,22]. Consequently, pharmacological plausibility cannot be established by comparing nominal in vitro concentrations with total plasma concentrations alone. Translational assessment should instead consider unbound exposure, tissue and intracellular concentrations, active metabolites, target potency or affinity, and, where available, direct evidence of target engagement or pharmacodynamic activity.

The same principle applies to technologies intended to improve phytochemical translation. Advanced delivery systems may increase absorption, systemic exposure, or tissue distribution, but improved bioavailability does not by itself establish improved therapeutic efficacy [23]. Likewise, molecular docking, molecular dynamics, artificial intelligence-based target prediction, and network modelling can refine target prioritisation, but their outputs remain dependent on structural assumptions, training data, model performance, and experimental validation [24,25]. These approaches are therefore most informative when combined with orthogonal biochemical, cellular, genetic, and target-engagement validation and interpreted alongside pharmacokinetic, pharmacodynamic, and clinical evidence where relevant [19,26].

Against this background, the present review develops an evidence-based framework for evaluating network pharmacology-guided phytochemical polypharmacology. Mechanistic claims are considered across six levels of evidence: (i) computationally predicted compound–target relationships; (ii) biochemical evidence of molecular interaction or inhibition; (iii) cellular target or pathway modulation; (iv) genetic, functional, or target-engagement evidence supporting causal contribution; (v) in vivo pharmacological and exposure evidence; and (vi) clinical pharmacodynamic or therapeutic evidence. This operational hierarchy is applied to curcumin, berberine, EGCG, and resveratrol to assess whether proposed multi-target mechanisms are experimentally supported and pharmacologically achievable.

The review further examines major factors that can disconnect network-level hypotheses from clinically relevant pharmacology, including exposure mismatch, metabolism, assay interference, pathway redundancy and compensation, microbiome-mediated transformation, dose–response complexity, and uncertainty surrounding direct target engagement. Particular attention is given to distinguishing pathway convergence from pharmacological synergy, association from causal mediation, adaptive responses from nonspecific stress or toxicity, and improved pharmacokinetic exposure from demonstrated therapeutic benefit.

Thus, rather than cataloguing large numbers of predicted targets or signalling pathways, this review adopts a critical translational perspective in which network pharmacology is used to prioritise mechanistic hypotheses, with pharmacological credibility contingent on exposure-compatible target engagement, causal validation, and evidence of biological or clinical relevance.

Figure 1 summarises the representative phytochemicals, principal signalling networks, evidentiary framework, translational barriers, and therapeutic strategies considered in this review.

## 2. Literature Search Strategy, Study Selection, and Evidence Appraisal

This article was designed as a structured narrative review rather than a systematic review or quantitative meta-analysis. The literature search was undertaken to identify representative and methodologically informative evidence relevant to phytochemical polypharmacology, network pharmacology, molecular mechanisms, pharmacokinetics, target validation, clinical translation, and advanced drug-delivery strategies. Because the review was not conducted according to a prospectively registered systematic review protocol, a PRISMA-style numerical accounting of retrieved, excluded, and included records was not maintained.

Literature searches were conducted in PubMed/MEDLINE, Scopus, and Web of Science, covering publications available through August 2026. Searches combined terms related to the pharmacological framework, representative phytochemicals, molecular mechanisms, pharmacokinetics, experimental validation, clinical translation, and emerging technologies.

The principal search terms included “polypharmacology”, “network pharmacology”, “systems pharmacology”, “phytochemical*”, “natural product*”, “curcumin”, “berberine”, “epigallocatechin gallate”, “EGCG”, “resveratrol”, “molecular target”, “target engagement”, “NF-κB”, “Nrf2”, “Keap1”, “AMPK”, “mTOR”, “STAT3”, “pharmacokinetic*”, “bioavailability”, “metabolite*”, “gut microbiome”, “drug delivery”, “nanoparticle*”, “phytosome*”, “PBPK”, “artificial intelligence”, “machine learning”, “CRISPR”, “single-cell”, and “spatial transcriptomics”. Terms were combined using the Boolean operators AND and OR, with database-specific syntax applied where appropriate.

A representative core search structure was:

(“polypharmacology” OR “network pharmacology” OR “systems pharmacology”) AND (“phytochemical*” OR “natural product*” OR “curcumin” OR “berberine” OR “epigallocatechin gallate” OR “EGCG” OR “resveratrol”).

This broad search was supplemented by compound-specific searches combining each phytochemical with mechanistic and translational terms such as “target engagement”, “binding”, “NF-κB”, “Nrf2”, “AMPK”, “STAT3”, “pharmacokinetic*”, “bioavailability”, “metabolite*”, “clinical trial”, “microbiome”, and “drug delivery”. Additional targeted searches addressed methodological topics central to the review, including molecular docking, receptor flexibility, artificial intelligence-based target prediction, CRISPR-based functional validation, cellular target-engagement methods, single-cell and spatial omics, and PBPK modelling. Reference lists of relevant primary studies, reviews, and methodological papers were also examined to identify additional literature pertinent to specific mechanistic or translational questions.

Curcumin, berberine, EGCG, and resveratrol were selected as representative case studies rather than as an exhaustive set of phytochemicals. They were chosen because the available literature for each compound spans multiple levels of evidence, including molecular and pathway-level pharmacology, pharmacokinetic characterisation, mechanistic investigation, and translational or clinical evaluation. This allowed comparison of different evidentiary levels within a common exposure-aware framework.

### 2.1. Study Selection

Study selection was based on relevance to the review’s predefined conceptual domains rather than on a formal systematic review eligibility algorithm. Studies were considered particularly informative when they addressed one or more of the following: compound–target interaction, biochemical activity, cellular pathway modulation, target engagement, genetic or functional validation, pharmacokinetics or metabolism, tissue exposure, microbiome-mediated transformation, human pharmacodynamic effects, clinical outcomes, drug-delivery strategies, or computational methods relevant to phytochemical target prioritisation.

For mechanistic studies, greater weight was given to investigations that clearly specified the tested compound, experimental model, concentration or dose, and molecular or functional readout. Evidence based on biochemical interaction measurements, cellular target engagement, genetic or pharmacological perturbation, rescue experiments, resistant mutants, or other orthogonal approaches was considered stronger than evidence based solely on downstream pathway modulation. This distinction is consistent with contemporary approaches to target identification and mechanism-of-action validation [19,26].

For pharmacokinetic studies, preference was given to reports that described a defined dose and formulation, together with quantitative measurements of the parent compound and/or relevant metabolites. Human pharmacokinetic and tissue exposure studies were prioritised in assessing translational plausibility. Total, unconjugated, and unbound exposures were distinguished where the source data permitted.

For clinical evidence, randomised controlled trials, prospective intervention studies, systematic reviews, and meta-analyses were preferentially considered. Clinical efficacy, pharmacodynamic responses, surrogate or intermediate endpoints, and safety or tolerability were treated as distinct categories of evidence.

Computational and network-pharmacology studies were included when relevant to target prediction, docking, network construction, pathway enrichment, artificial intelligence, or systems-level hypothesis generation. However, computational predictions without corresponding biochemical, cellular, functional, pharmacokinetic, or target-engagement validation were treated as hypothesis-generating rather than as confirmation of the molecular mechanism [19,24].

Primary studies were preferentially used for specific quantitative statements, including binding or inhibition constants, human pharmacokinetic parameters, tissue concentrations, target-engagement findings, and clinical outcomes. Reviews and meta-analyses were used primarily to contextualise broader bodies of evidence.

Non-English publications, conference abstracts lacking sufficient methodological detail, non-peer-reviewed reports, and studies lacking sufficient experimental information to evaluate the claim of interest were generally not used as primary evidence. Articles outside the mechanistic or translational scope of the review were likewise not considered in detail.

### 2.2. Evidence Appraisal

Evidence was evaluated based on the type and strength of experimental support rather than on the number of publications reporting an association. Compound–target relationships were distinguished among computational prediction, biochemical interaction or inhibition, cellular pathway modulation, cellular target engagement, functional or genetic evidence, in vivo pharmacology, and human pharmacokinetic, pharmacodynamic, or clinical evidence [19,26].

Particular attention was given to the correspondence between experimental potency or concentration, achievable human or tissue exposure, target engagement, and functional response. Computational prediction or pathway enrichment alone was not regarded as evidence of direct molecular engagement. Similarly, convergence of several phytochemicals on the same signalling pathway was not interpreted as evidence of pharmacological synergy without appropriate quantitative interaction analysis [27].

Where findings differed among studies, discrepancies were considered in relation to experimental model, dose or concentration, compound purity, formulation, exposure duration, analytical methodology, pharmacokinetic context, and strength of target validation.

Because this review was conducted as a structured narrative synthesis, formal risk-of-bias scoring, protocol registration, quantitative meta-analysis, pooled effect estimation, and PRISMA-style study-flow accounting were not performed. This methodological limitation is acknowledged explicitly to avoid implying the completeness or reproducibility expected of a systematic review.

The objective was therefore to provide a critical, mechanistically integrated, and translationally focused synthesis of representative evidence, rather than an exhaustive systematic catalogue of all publications in the field.

## 3. Systems-Level Foundations of Phytochemical Polypharmacology

### 3.1. Network Pharmacology and Disease Modules

Many complex disease phenotypes arise from perturbations of interconnected gene and protein subnetworks, often conceptualised as disease modules, rather than from dysfunction of a single molecular target [3,28]. This systems-level perspective provides the conceptual basis for network pharmacology, which integrates compound–target associations, protein–protein interaction (PPI) networks, and functional or pathway enrichment analyses to generate hypotheses concerning the molecular basis of complex therapeutic responses [6,7]. This framework is particularly relevant to natural products and traditional medicines, in which multiple constituents may interact with partially overlapping biological systems [6,11].

Network-pharmacology analyses of medicinal plants and phytochemicals, including *Ganoderma* species, have reported associations with inflammatory, metabolic, oxidative-stress, and survival-related pathways, including NF-κB, PI3K/Akt, MAPK, Nrf2, and AMPK/mTOR signalling [7,29]. Such analyses can prioritise biologically plausible targets and pathways for experimental investigation, but neither pathway enrichment nor network connectivity establishes direct target engagement, simultaneous modulation of multiple proteins, or causal pathway involvement [5]. Accordingly, network-level associations are interpreted in this review as mechanistic hypotheses rather than established pharmacological mechanisms unless supported by independent experimental evidence.

PPI networks can be subjected to topological analysis to identify highly connected nodes, or “hubs”, within disease-associated networks [5]. Such nodes may provide useful candidates for experimental investigation. However, network centrality should not be equated with pharmacological importance because network representations depend on database coverage, interaction evidence, biological context, and analytical assumptions [5]. A highly connected node in a static network is therefore not necessarily an accessible drug target or a functionally dominant mediator of a phytochemical response.

Functional enrichment analyses provide an additional layer of interpretation by assessing whether predicted or experimentally identified target sets are statistically associated with specific biological processes or pathways, as represented by Gene Ontology (GO) and Kyoto Encyclopedia of Genes and Genomes (KEGG) annotations [5]. Their interpretation depends on the input target set, the database, the annotation coverage, and the analytical parameters. Overlap among related biological pathways may consequently contribute to apparent network convergence without demonstrating independent pharmacological modulation. Enrichment analyses should therefore be used primarily to prioritise biological hypotheses rather than establish pathway-level mechanisms [5].

The apparent complexity of a predicted network should likewise not be equated with the pharmacological complexity of a compound. Broad pathway modulation may arise from a smaller number of primary molecular events, whereas current databases or prediction algorithms may incompletely represent genuine multi-target effects. Network pharmacology is therefore most appropriately used to organise and prioritise hypotheses for subsequent experimental validation [5,19].

### 3.2. Evidentiary Hierarchy: From Computational Prediction to Validated Polypharmacology

A central premise of this review is that network convergence is not equivalent to demonstrated polypharmacology. Shared enrichment of NF-κB, AMPK, Nrf2, PI3K/Akt, or related pathways may reflect engagement of multiple primary targets, downstream convergence following perturbation of fewer upstream targets, adaptive cellular responses, or biases arising from prediction algorithms, database coverage, network topology, and pathway annotation. These possibilities cannot be reliably distinguished from network topology or enrichment analysis alone [5].

For this review, heterogeneous mechanistic evidence is classified using the following operational six-tier framework.

**Tier 0—Computational/predicted evidence.** Molecular docking, network pharmacology, quantitative structure–activity relationship modelling, molecular dynamics, knowledge-graph analysis, or AI-based target prediction without independent biochemical or cellular confirmation. Such evidence is considered hypothesis-generating.

**Tier 1—Biochemical/direct molecular evidence.** Cell-free binding, enzymatic inhibition or activation, and covalent-modification studies performed under defined experimental conditions, including surface plasmon resonance, isothermal titration calorimetry, and validated biochemical activity assays. Depending on the assay, these approaches provide evidence of molecular interaction or biochemical activity, but do not by themselves establish cellular or physiological relevance [19].

**Tier 2—Cellular/pathway evidence.** Cell-based reporter assays, phosphorylation or transcriptional measurements, target-expression studies, and phenotypic experiments demonstrating modulation of a molecular pathway or cellular process. Such studies establish cellular activity but do not necessarily identify the molecular event responsible for the observed response [19].

**Tier 3—Functional/causal and target-engagement evidence.** Genetic perturbation approaches, including CRISPR knockout/knock-in, knockdown, rescue, or resistant-mutant studies, together with orthogonal target-engagement methods such as cellular thermal shift assays (CETSA) or chemoproteomic approaches [26,30,31]. Genetic dependency and physical target engagement provide complementary rather than interchangeable evidence. Confidence in target attribution is greatest when independent functional and target-engagement approaches converge on the same proposed mechanism [19,26].

**Tier 4—In vivo/animal evidence**—demonstration of pharmacological, biomarker, or phenotypic effects in an intact organism. Interpretation requires consideration of species differences, dose, formulation, route of administration, metabolism, tissue distribution, and exposure.

**Tier 5—Clinical evidence.** Evidence obtained in humans, including pharmacodynamic responses, validated biomarkers, clinical endpoints, or therapeutic outcomes. Its strength depends on study design, sample size, formulation, exposure, endpoint validity, reproducibility, and the distinction between pharmacodynamic activity and demonstrated clinical benefit. These tiers distinguish types of evidence rather than impose a rigid linear ranking. A biochemical interaction may provide stronger evidence of direct molecular interaction than a downstream clinical biomarker. In contrast, a human pharmacodynamic response may demonstrate biological activity without identifying the responsible molecular target. The framework, therefore, separates what has been directly demonstrated from what remains predicted, inferred, or causally unresolved.

Throughout Section 4 and Section 5, mechanistic claims are interpreted according to this framework. Where evidence of target-specific perturbation or direct target engagement is unavailable, pathway-level associations are described as putative or hypothesis-generating rather than as established molecular mechanisms.

### 3.3. Selective Non-Selectivity: Defining the Therapeutic Window of Polypharmacology

The concept of “selective non-selectivity”, developed in the context of multi-target drug design, describes an approach in which activity across several therapeutically relevant targets is balanced against avoidance of pharmacologically undesirable interactions [32]. It therefore provides a useful conceptual distinction between purposeful multi-target pharmacology and indiscriminate molecular promiscuity. Importantly, however, the presence of several measurable molecular interactions does not by itself establish therapeutically useful selectivity [10,32].

Affinity or potency alone is insufficient to determine whether a multi-target interaction profile is pharmacologically meaningful. For this review, reported IC_50_, K_i_, Kd, or EC_50_ values are interpreted in relation to unbound drug concentration, tissue distribution, target abundance, binding kinetics (where known), cellular permeability, subcellular localisation, active metabolites, and the pharmacodynamic consequences of target modulation. Potency measured in a purified biochemical system may differ substantially from activity within cellular or tissue environments.

This issue is particularly relevant to phytochemicals because individual compounds are frequently associated with the modulation of several molecular targets and signalling pathways [7]. Such breadth should not automatically be interpreted as pharmacologically advantageous. For chemically reactive or assay-interfering compounds, apparent broad activity may also arise from nonspecific reactivity, aggregation, redox-related assay effects, or other assay-interference mechanisms [12,33,34]. The pharmacological value of a multi-target profile, therefore, depends on which targets are engaged, at what exposure, in which tissues, for what duration, and with what functional consequences.

For this critical review, therapeutically credible polypharmacology is operationally evaluated using five criteria:

1. **Exposure sufficiency.** The proposed interaction should be compatible with pharmacologically relevant exposure at the site of action, with particular attention to unbound concentrations, tissue and intracellular distribution, and active metabolites. Comparisons between target potency and systemic exposure should be interpreted cautiously when local concentrations are unknown.
2. **Target engagement and pharmacodynamic concordance.** Where feasible, evidence should demonstrate engagement of the proposed target under relevant experimental conditions and show that target modulation corresponds temporally and quantitatively with an appropriate pharmacodynamic response.
3. **Causal or functional linkage.** The proposed target should have a demonstrable relationship to the phenotype, supported where possible by genetic perturbation, selective pharmacological inhibition, rescue, resistant-mutant studies, or related functional approaches.
4. **Therapeutic selectivity across the target network.** Multi-target activity should provide a plausible therapeutic advantage without disproportionate engagement of unrelated or counterproductive targets at achievable exposure.
5. **Perturbation concordance.** Independent perturbation of individual proposed targets, or rational combinations of targets, should reproduce or modify at least part of the phenotype attributed to the phytochemical, strengthening the inference that the proposed target set contributes causally rather than correlatively.

Based on the evidence evaluated in this review, none of the four representative phytochemicals consistently satisfies all five criteria across their broad reported target profile. Berberine provides comparatively strong evidence for an energy-sensing, AMPK-associated pharmacological axis, including mechanisms involving mitochondrial complex I and downstream metabolic signalling. However, its low systemic parent-drug exposure and tissue-specific pharmacokinetics remain important translational considerations. By contrast, many reported cellular effects of curcumin and EGCG occur at concentrations that may exceed clinically achievable free systemic exposure, although local exposure, formulation, metabolism, and metabolite activity complicate direct comparison [16,20,21,35]. Accordingly, therapeutically useful selectivity remains a compound-, target-, tissue-, and exposure-dependent empirical question, rather than a conclusion that can be inferred from the breadth of a reported target list.

### 3.4. Hormesis and Non-Monotonic Dose–Response Relationships

Hormetic dose–response relationships have been extensively reported for resveratrol, with lower and higher exposures producing qualitatively different biological effects in multiple experimental systems [36]. More generally, hormesis is defined as an adaptive response characterised by a biphasic dose–response relationship [37]. However, not every non-monotonic or biphasic dose–response relationship should automatically be classified as hormesis. Apparent non-monotonic responses may also be influenced by cytotoxicity, compound aggregation, nonspecific chemical reactivity, redox-related assay effects, or other forms of assay interference [33,34].

For this review, a response is classified as hormetic when it demonstrates: (i) a reproducible biphasic dose–response relationship; (ii) a low-dose stimulatory response compatible with an adaptive biological process; (iii) evidence that the stimulatory phase is not simply a consequence of compromised viability or nonspecific toxicity; and (iv) appropriate experimental controls and replication supporting a genuine adaptive response rather than an assay-specific artefact [36,37].

For example, activation of Keap1–Nrf2–antioxidant response element signalling can participate in adaptive cellular responses to electrophilic or oxidative stress. However, increased Nrf2-associated transcription alone does not establish hormesis; an appropriate dose–response relationship and separation of adaptive signalling from toxicity are also required [38,39].

This distinction is particularly important for chemically reactive phytochemicals. At increasing experimental concentrations, apparent changes in activity may be influenced by redox chemistry, nonspecific chemical reactivity, aggregation, assay interference, or overt cytotoxicity [12,33,34]. Such phenomena can produce concentration-dependent or non-monotonic responses without demonstrating a therapeutically beneficial hormetic mechanism. The terms hormesis, non-monotonic dose–response, and dose-dependent toxicity should therefore not be used interchangeably [37].

From a translational perspective, this review further considers a hormetic mechanism clinically relevant only when the adaptive response occurs within a pharmacologically achievable exposure range, produces a reproducible pharmacodynamic effect, and is not outweighed by toxicity or nonspecific molecular effects. A low concentration that activates a stress-response pathway and a higher concentration that reduces cellular viability are not, in themselves, sufficient to establish a therapeutically meaningful hormetic mechanism.

Taken together, these principles establish the conceptual framework used throughout this review. Network pharmacology prioritises candidate targets and pathways; biochemical and cellular studies characterise molecular and pathway activity; functional perturbation and target-engagement studies strengthen causal attribution; PK/PD analyses assess whether proposed mechanisms are compatible with in vivo exposure and response; and clinical studies evaluate whether such pharmacology translates into meaningful human benefit. The resulting translational workflow, incorporating iterative computational prediction, experimental validation, pharmacokinetic/pharmacodynamic assessment, formulation optimisation, biomarker evaluation, and clinical testing, is summarised in Figure 2.

## 4. Molecular Architecture and Polypharmacological Profiles of Representative Phytochemicals

### 4.1. Molecular Target Landscape and Network Interactions

Curcumin, berberine, epigallocatechin-3-gallate (EGCG), and resveratrol are widely investigated phytochemicals associated with inflammatory, metabolic, redox-sensitive, and cell-survival signalling processes [7]. However, their chemical structures, physicochemical properties, pharmacokinetic behaviour, and levels of mechanistic evidence differ substantially [12,14,21,40]. Accordingly, the target profiles considered below distinguish direct or putative biochemical interactions (Tier 1) from cellular pathway effects (Tier 2) and, where available, functional, causal, or target-engagement evidence (Tier 3). Reported target associations are therefore not assumed to represent equivalent pharmacological mechanisms.

1. Curcumin (diferuloylmethane) is a diarylheptanoid containing a conjugated β-diketone system and electrophilic α,β-unsaturated carbonyl functionality capable of reacting with nucleophilic residues under appropriate experimental conditions [12,41]. Curcumin has also been associated with modulation of NF-κB-, STAT3-, PI3K/Akt-, and other kinase-related signalling pathways in cellular systems [42,43]. These observations should not be interpreted uniformly as evidence of direct target engagement. Biochemical interaction or inhibition constitutes Tier 1 evidence. In contrast, suppression of NF-κB-, STAT3-, or related signalling in cells generally constitutes Tier 2 evidence unless target-specific perturbation or orthogonal target-engagement approaches establish a causal molecular relationship. Curcumin’s electrophilic reactivity, chemical instability, and assay-interference liability further complicate assignment of broad cellular phenotypes to individual molecular targets [12]. Experimentally observed molecular interactions should therefore be distinguished from evidence that those interactions occur at pharmacologically relevant exposure and causally contribute to therapeutic effects.
2. Berberine is an isoquinoline alkaloid whose metabolic actions have been linked experimentally to functional inhibition of mitochondrial respiratory complex I, altered cellular energy status, and subsequent activation of AMP-activated protein kinase (AMPK) [44]. This complex I–energy-sensing–AMPK pathway represents a well-characterised experimental pharmacological axis associated with berberine. Importantly, the available evidence supports functional inhibition of mitochondrial respiration rather than establishing direct binding to a specific molecular subunit of complex I, and AMPK activation is best interpreted primarily as a downstream cellular response rather than as evidence that AMPK itself is a direct molecular target of berberine.

Berberine also affects broader metabolic signalling and undergoes substantial interaction with intestinal, metabolic, and microbial processes [14,44,45]. These effects require separate consideration because their interpretation depends on intestinal and tissue exposure, metabolism, microbial transformation, and evidence of causal mediation. Berberine, therefore, illustrates how a comparatively well-characterised pharmacological pathway can coexist with a broader set of reported biological effects whose directness, exposure compatibility, and translational relevance remain variable.

3. EGCG is a catechin polyphenol containing multiple hydroxyl groups and a gallate moiety, and is susceptible to oxidation under experimental conditions [16,21]. A seminal biochemical study identified the 67 kDa laminin receptor (67LR) as an EGCG-binding protein using surface plasmon resonance, providing direct biophysical evidence of molecular interaction [46]. The contribution of this interaction to biological responses nevertheless remains dependent on cellular context, exposure, and downstream functional validation.

EGCG has also been reported to inhibit DNA methyltransferase (DNMT) activity in a cell-free nuclear-extract assay, with competitive inhibition observed at micromolar concentrations [17]. Because the assay used nuclear extracts rather than purified DNMT1, these data support biochemical inhibition of DNMT activity but do not by themselves establish selective direct engagement of DNMT1. EGCG-associated modulation of PI3K/Akt, MAPK, NF-κB, Nrf2, and receptor-associated signalling has predominantly been demonstrated at the cellular level [7,21]. Such observations therefore support pathway modulation rather than direct engagement of every molecular component within these pathways. EGCG auto-oxidation and formation of reactive products further complicate the interpretation of experiments performed at relatively high concentrations [16,21].

4. Resveratrol (3,5,4′-trihydroxy-*trans*-stilbene) was historically proposed to directly activate Sirtuin 1 (SIRT1). Subsequent work challenged this interpretation by demonstrating that apparent SIRT1 activation could depend on fluorophore-containing assay substrates and was not consistently reproduced using native substrates [18]. The resveratrol–SIRT1 relationship is therefore more appropriately described as SIRT1-associated or mechanistically disputed rather than as an unequivocally established direct molecular interaction [18].

Several effects previously attributed to direct SIRT1 activation may instead involve indirect mechanisms. Experimental studies have implicated inhibition of cyclic-AMP phosphodiesterases, altered cAMP signalling, CaMKKβ-dependent AMPK activation, and downstream NAD^+^/SIRT1-associated signalling [47]. Resveratrol is also associated with broader metabolic, inflammatory, and stress-response pathways [7,47]. These findings illustrate why downstream signalling responses should not be regarded as equivalent to validated direct target engagement.

Taken together, the four phytochemicals illustrate distinct forms of mechanistic complexity. Curcumin combines electrophilic reactivity with broad cellular signalling effects; berberine possesses a comparatively well-characterised energy-sensing pharmacological axis alongside additional metabolic and intestinal mechanisms; EGCG combines experimentally supported molecular interactions with chemical instability and exposure constraints; and resveratrol has a historically prominent but disputed SIRT1 mechanism alongside indirect signalling routes. Their inclusion as representative case studies does not imply equivalent or fully validated polypharmacological profiles. Rather, they illustrate how predicted or reported target networks should be evaluated against biochemical, cellular, functional, pharmacokinetic, in vivo, and clinical evidence.

A compound-centred translational comparison is provided in Table 1.

### 4.2. Redox Switching and Context-Dependent Stress Responses

Curcumin and EGCG exhibit chemical and redox reactivity under experimental conditions [12,16,41], whereas berberine can alter cellular redox and metabolic states through effects on mitochondrial energy metabolism [44]. Resveratrol has also been associated with dose-dependent adaptive and stress-related responses [36]. However, describing all four compounds as exhibiting a uniform physiologically meaningful “redox switch” would overstate the available evidence.

Curcumin can undergo oxidation and electrophilic reactions associated with its conjugated carbonyl system, whereas EGCG is particularly susceptible to auto-oxidation [12,16,41]. Under appropriate experimental conditions, such chemistry can generate reactive intermediates and influence redox-sensitive signalling. Electrophilic or oxidative stress may activate cytoprotective signalling through the Keap1–Nrf2 pathway [38,39]. These observations are more appropriately interpreted as context-dependent biochemical and cellular effects (Tier 1–2) rather than as evidence of a universal physiological antioxidant mechanism.

Activation of Nrf2-associated transcription alone should not be interpreted as evidence of direct Keap1 modification or hormesis. Demonstration of direct target modification requires appropriate biochemical or target-engagement evidence, whereas classification as hormesis additionally requires a reproducible biphasic dose–response relationship and separation of adaptive responses from nonspecific toxicity [37].

Berberine lacks the polyphenolic architecture characteristic of EGCG and curcumin. Its effects on cellular stress and redox balance are more appropriately considered in relation to mitochondrial respiratory complex I inhibition, altered cellular energy metabolism, and downstream signalling [44]. Resveratrol-associated metabolic and stress responses may likewise involve indirect cAMP–CaMKKβ–AMPK and NAD^+^/SIRT1-associated signalling [47].

The apparent antioxidant or pro-oxidant behaviour of phytochemicals should therefore be interpreted as exposure- and context-dependent pharmacology rather than an intrinsic binary property. For compounds with known chemical reactivity or assay interference liabilities, high experimental concentrations may increase the contribution of nonspecific chemical reactivity, aggregation, assay interference, or cytotoxicity to the observed phenotypes [12,33,34]. Redox-associated pathway activation should consequently be assigned mechanistic significance only when supported by appropriate biochemical, cellular, functional, or target-engagement evidence.

### 4.3. Epigenetic Modulation

Epigenetic effects have been proposed as an additional component of phytochemical activity. Still, direct enzyme inhibition should be distinguished from downstream changes in DNA methylation, histone modification, chromatin regulation, and non-coding RNA expression.

EGCG has been reported to inhibit DNMT activity in a cell-free nuclear-extract assay at micromolar concentrations [17]. Because this assay used nuclear extracts rather than purified DNMT1, the results support biochemical inhibition of DNMT activity but do not by themselves establish selective direct engagement of DNMT1. The same study reported reactivation of several methylation-silenced genes in cellular models [17]. These findings constitute cellular epigenetic evidence (Tier 2) rather than proof of clinically relevant DNMT1 engagement.

Curcumin has been shown to inhibit p300/CBP-associated histone acetyltransferase activity in biochemical systems [54]. Separately, curcumin has been reported to alter microRNA expression profiles in human pancreatic cancer cells [55]. These observations represent different levels of evidence: biochemical enzyme inhibition constitutes direct molecular evidence under defined experimental conditions, whereas changes in microRNA expression reflect cellular regulatory responses. Altered microRNA expression should therefore not be interpreted as evidence that individual microRNAs are direct molecular targets of curcumin.

Resveratrol has been associated with SIRT1-related deacetylation and mitochondrial regulation, but interpretation remains constrained by uncertainty regarding direct SIRT1 activation [18,47]. These effects are therefore better described as SIRT1- or AMPK-associated downstream responses than as unequivocal evidence of direct resveratrol–SIRT1 engagement.

Overall, epigenetic observations should not be incorporated into a polypharmacological target network solely because changes in DNA methylation, histone modification, chromatin state, or gene expression are observed. Stronger target attribution requires evidence connecting the phytochemical to a defined molecular target, demonstrating target engagement where feasible, and establishing that target modulation contributes functionally to the observed phenotype [19,26].

### 4.4. Microbiome-Mediated Indirect Pharmacology: Association Versus Causation

Curcumin, EGCG, and berberine exhibit limited systemic exposure to their unmetabolized parent forms after conventional oral administration, while gastrointestinal exposure, metabolism, and microbial transformation may contribute substantially to their disposition and biological effects [14,16,45,56]. These characteristics have stimulated interest in the gut microbiome as a potential contributor to the pharmacology of phytochemicals. However, microbiome-associated changes should not automatically be interpreted as evidence that microbial modulation mediates a therapeutic phenotype.

The evidence for berberine illustrates this distinction. In the PREMOTE study, berberine treatment was associated with changes in gut microbial composition, bile-acid metabolism, and metabolic outcomes, with mechanistic analyses implicating altered microbial bile-acid biotransformation involving *Ruminococcus bromii* [57]. Nevertheless, concurrent changes in microbial composition and host metabolic endpoints do not establish that every altered microbial population is necessary or sufficient for the therapeutic effect.

Causal mediation requires experimental approaches that can separate microbial effects from parallel host- or drug-mediated mechanisms. Relevant strategies include germ-free or gnotobiotic models, defined microbial communities, faecal microbiota transplantation, targeted manipulation of candidate organisms, and administration of defined microbial metabolites [58]. Convergent evidence from such approaches provides stronger support for microbiome-mediated pharmacology than changes in microbial abundance alone.

Comparable caution applies to curcumin. A small randomised pilot study reported changes in gut microbial diversity and composition following turmeric or curcumin supplementation, but responses were highly individualised [59]. These findings demonstrate microbiome responsiveness to treatment but do not establish that particular microbial taxa causally mediate therapeutic effects.

An additional complication is that the pharmacologically relevant molecular species may differ from the administered parent compound. Gut bacteria can convert berberine into a more readily absorbable form, demonstrating that microbial metabolism can alter its intestinal disposition [45]. Parent compounds, host-generated metabolites, and microbiota-derived metabolites should therefore be considered separately when interpreting phytochemical pharmacology [45,60].

Accordingly, microbiome-mediated effects are considered here as an indirect layer of pharmacology rather than as evidence of polypharmacology per se. Host–microbiome–metabolite interactions may contribute to biological responses, but demonstrating changes in multiple microbes or hosts does not establish simultaneous, therapeutically relevant engagement of multiple molecular targets. Limited systemic exposure of a parent compound does not necessarily exclude intestinal or metabolite-mediated pharmacology; conversely, therapeutic effects should not be attributed to “microbiome modulation” solely because microbial composition changes.

The microbiome is therefore best incorporated into phytochemical network models as a candidate intermediate biological layer between oral exposure and host molecular effects rather than automatically as an established therapeutic target. Demonstration of causal mediation requires integrating microbiome perturbation, metabolite measurements, pharmacokinetics, target engagement (where applicable), and phenotypic outcomes.

## 5. Major Signalling Pathways and Network Convergence

Throughout this section, pathway associations are interpreted according to the evidentiary hierarchy defined in Section 3.2. Computational and network-level associations are considered hypothesis-generating; cellular pathway modulation is distinguished from direct molecular engagement; and causal claims are reserved for evidence supported by functional, genetic, pharmacological, or target-engagement studies. Terms such as “synergy” and “emergent effect” are used only when supported by appropriate quantitative or mechanistic evidence.

### 5.1. NF-κB Pathway

NF-κB signalling is a frequently reported point of pathway-level convergence among curcumin, berberine, EGCG, and resveratrol, although their molecular entry points differ substantially [7].

Curcumin has been reported to suppress IKK/NF-κB-associated signalling in cellular systems [42,43]. However, these observations should not automatically be interpreted as evidence that IKKβ is the dominant intracellular target responsible for NF-κB suppression. Curcumin’s electrophilic reactivity, chemical instability, and assay-interference liability complicate attribution of broad cellular effects to a single molecular interaction [12]. Accordingly, cellular NF-κB suppression is classified primarily as Tier 2 evidence unless supported by direct target-engagement and functional studies.

EGCG has been shown to bind the 67 kDa laminin receptor (67LR) and has independently been reported to suppress NF-κB-associated signalling in cellular systems [21,46]. However, the presence of a validated EGCG–67LR interaction does not by itself establish that 67LR is the dominant mediator of NF-κB suppression. The NF-κB phenotype is therefore best interpreted primarily as modulation of cellular pathways (Tier 2), based on the evidence in the present bibliography.

Berberine inhibits mitochondrial respiratory complex I and subsequently activates AMPK-associated energy-sensing pathways [44]. NF-κB-associated modulation has also been reported for berberine [7], but the available references do not establish a single causal complex I–AMPK–NF-κB pathway. The relationship is therefore more appropriately regarded as indirect pathway-level convergence rather than direct NF-κB engagement.

SIRT1 can repress NF-κB transcriptional activity by deacetylating RelA/p65 at Lys310, and resveratrol treatment was associated with reduced NF-κB-dependent transcription in the original cellular study [61]. However, direct activation of SIRT1 by resveratrol is mechanistically disputed, whereas alternative studies support indirect PDE–cAMP–AMPK–NAD^+^/SIRT1-associated signalling. Resveratrol-mediated NF-κB suppression should therefore be described as a SIRT1-associated cellular mechanism rather than evidence of direct resveratrol–SIRT1 engagement [18,47].

Collectively, these observations demonstrate pathway-level convergence without equivalent molecular mechanisms. Curcumin, berberine, EGCG, and resveratrol can influence NF-κB-associated signalling through different biochemical or cellular routes [7]. Shared suppression of the same downstream pathway does not, by itself, establish simultaneous multi-target engagement or an equivalent causal mechanism.

Accordingly, NF-κB suppression is not treated in this review as intrinsically beneficial; its pharmacological significance is considered to depend on the biological and disease context.

### 5.2. Nrf2/Keap1 Axis

The Nrf2/Keap1 system is another frequently reported signalling axis associated with phytochemical responses. However, the mechanisms leading to Nrf2 activation differ among compounds and should not be reduced to a generalised antioxidant effect [7,39].

Curcumin contains electrophilic structural features capable of reacting with nucleophilic residues [41], whereas EGCG is susceptible to oxidation and formation of reactive products under experimental conditions [16]. More generally, electrophilic or oxidative modification of redox-sensitive Keap1 cysteines can disrupt Keap1-dependent repression of Nrf2 and promote antioxidant-response-element-dependent transcription [38,39]. These chemical- and pathway-level observations, however, do not demonstrate that curcumin or EGCG directly engages Keap1 under pharmacologically relevant intracellular conditions.

Cellular Nrf2 activation should therefore be distinguished from direct Keap1 engagement. Demonstrating a specific phytochemical–Keap1 interaction requires biochemical or target-engagement evidence, whereas demonstrating that such an interaction causes a biological phenotype requires additional functional validation.

Nrf2 activation should likewise not automatically be described as hormetic. As defined in Section 3.4, hormesis requires an appropriately characterised biphasic dose–response relationship rather than pathway activation alone [37].

Nrf2 is functionally interconnected with inflammatory, metabolic, and stress-response networks, including NF-κB and kinase-associated signalling [39]. It is therefore appropriately considered a regulatory node within a broader signalling network, while the therapeutic relevance of its modulation remains dependent on exposure, tissue context, and causal evidence.

### 5.3. AMPK/mTOR/Autophagy

AMPK is a central cellular energy-sensing kinase that interacts directly with mTOR-regulated autophagy mechanisms, including regulation of ULK1 [62].

Among the four phytochemicals considered here, berberine has comparatively strong mechanistic evidence for mitochondrial respiratory complex I inhibition, followed by altered cellular energy status and AMPK activation [44]. However, systemic exposure to unchanged berberine in humans is low, and intestinal, tissue-specific, and metabolite-mediated exposure must be considered when extrapolating these mechanisms to clinical pharmacology [14].

Resveratrol can activate AMPK indirectly through PDE inhibition, increased cAMP signalling, CaMKKβ activation, and downstream metabolic signalling [47]. Curcumin has been reported to influence Akt/mTOR and autophagy-associated processes in cellular models [42], while EGCG has also been associated with cellular autophagy-related responses [21]. These observations predominantly represent Tier 2 pathway evidence and do not establish AMPK, mTOR, or the autophagy machinery as direct molecular targets of each compound.

AMPK can suppress mTORC1 activity and regulate autophagy through phosphorylation of ULK1 [62]. Nevertheless, observation of AMPK-, mTOR-, or autophagy-associated changes across several phytochemicals does not establish a shared causal mechanism. Similar downstream pathway changes can arise from distinct upstream molecular perturbations.

AMPK convergence should therefore be regarded as a plausible organising framework rather than a universal mechanism of phytochemical action. Stronger causal attribution requires pathway-specific or target-specific perturbation together with complementary molecular evidence [19,26].

### 5.4. STAT3 Signalling

Persistent STAT3 activation occurs in multiple malignancies and can contribute to tumour-cell proliferation, survival, inflammatory signalling, and immune regulation [63].

Curcumin has been reported to suppress JAK/STAT3-associated signalling in cellular cancer models [43]. Based on the references available here, this is best regarded as cellular pathway modulation (Tier 2) rather than as evidence of direct curcumin–STAT3 engagement.

EGCG has been shown experimentally to suppress STAT3 signalling in human pancreatic cancer models [64]. Resveratrol has likewise been shown to inhibit Src–STAT3 signalling and reduce STAT3-dependent transcriptional activity in malignant cells [65]. These studies support cellular inhibition of STAT3-associated signalling but do not demonstrate that EGCG and resveratrol engage STAT3 through identical molecular mechanisms.

The recurring observation across these compounds is therefore more accurately described as convergent suppression of STAT3-associated signalling rather than equivalent STAT3-targeted pharmacology. Reduced STAT3 phosphorylation or transcriptional activity does not by itself identify the initiating molecular target; direct target attribution requires orthogonal biochemical or target-engagement evidence [19,26].

### 5.5. Pathway Crosstalk: Additive, Cooperative, or Synergistic

The signalling pathways discussed above are interconnected. AMPK regulates mTOR and autophagy-associated signalling [62]. Nrf2 interacts with inflammatory and stress-response networks [39]; and SIRT1 can regulate NF-κB-dependent transcription through deacetylation of *RelA*/p65 [61]. These relationships provide a biological basis for the propagation of perturbations across signalling modules.

However, pathway crosstalk should not be equated with pharmacological synergy. Simultaneous modulation of multiple pathways can yield additive, synergistic, antagonistic, or compensatory responses, depending on the biological system and experimental conditions.

Formal synergy requires quantitative comparison with an explicit reference model. Approaches, including Bliss independence and Loewe additivity, can be selected according to the experimental design and underlying assumptions [27]. Consequently, simultaneous changes in multiple signalling pathways alone are insufficient to establish pharmacological synergy.

Similarly, this review does not use the term “emergent effect” merely because several pathways change simultaneously. The term is reserved for situations in which a network-level phenotype cannot be adequately accounted for by experimentally established individual effects or an appropriate additive framework.

Where formal interaction analysis is unavailable, the terms “network-level convergence” or “potentially cooperative effects” are used instead of “synergy”.

A pathway-centred comparison of the evidence supporting modulation of NF-κB, Nrf2/Keap1, AMPK/mTOR/autophagy, and STAT3 by curcumin, berberine, EGCG, and resveratrol is summarised in Table 2. The table distinguishes direct or functional molecular evidence from cellular pathway modulation, human pharmacodynamic observations, and conceptual network-level convergence.

### 5.6. Systems-Level Interpretation

Structurally distinct phytochemicals can produce overlapping pathway-level responses despite differing molecular entry points. Apparent convergence may arise from distinct primary molecular interactions, downstream signalling propagation, adaptive responses, or limitations of network and pathway analyses [7,13,62,63]. Accordingly, shared pathway annotations across curcumin, berberine, EGCG, and resveratrol should be regarded as candidate mechanistic intersections rather than evidence of equivalent polypharmacology.

Within the evidentiary framework defined in Section 3.2, these intersections should be prioritised for experimental testing, with translational relevance determined by appropriate molecular or functional support and compatibility with pharmacologically relevant exposure [19,26].

## 6. Pharmacokinetic Barriers and Translational Challenges

### 6.1. Quantitative Human Exposure Assessment

A central barrier to the clinical translation of phytochemical polypharmacology is the potential mismatch between the concentrations that produce biological effects in experimental systems and the exposures achievable in humans. This issue is particularly important for network pharmacology because identification of a predicted molecular target does not establish that a pharmacologically active species reaches that target at sufficient concentration in vivo or that target engagement occurs. Curcumin, berberine, EGCG, and resveratrol all exhibit substantial pharmacokinetic constraints involving absorption, distribution, metabolism, and/or formulation dependence that complicate direct extrapolation of mechanistic in vitro observations to human pharmacology [14,20,21,22].

Accordingly, Table 3 emphasises measured human parent-compound exposure, metabolites, tissue concentrations where available, and representative target-specific activity or affinity data. Unbound target-site concentrations are not inferred from total or unconjugated plasma concentrations when such measurements are unavailable.

The quantitative comparisons in Table 3 demonstrate why plasma concentration, tissue concentration, mechanistic activity concentration, and target affinity should not be treated as interchangeable quantities. For curcumin, a recent independent crossover study found very low unconjugated plasma concentrations even with formulations designed to enhance systemic exposure [67], whereas direct sampling in patients demonstrated measurable parent compound in colorectal tissue following oral administration [48]. Similarly, direct human tissue studies have detected EGCG and its methylated metabolite in prostate tissue and resveratrol together with multiple conjugated metabolites in colorectal tissue [51,52]. Low systemic parent-compound exposure, therefore, does not exclude local pharmacology, but tissue detection alone does not establish engagement of a particular molecular target.

EGCG illustrates why translational assessment should be target-specific rather than compound-wide. The reported affinity of EGCG for 67LR is in the nanomolar range, whereas inhibition of nuclear DNMT activity was observed at micromolar concentrations in a nuclear-extract assay. The latter study did not selectively establish purified DNMT1 engagement [17,46]. Thus, a human exposure that may be more consistent with one proposed molecular interaction cannot automatically be used to support another mechanism that requires substantially higher experimental concentrations.

A further limitation is the scarcity of quantitative unbound target-site concentrations. Total, unchanged, or unconjugated plasma measurements should not automatically be equated with the freely available concentration at an intracellular molecular target. Protein binding, membrane permeability, subcellular distribution, metabolite formation, exposure duration, and tissue-specific disposition can all affect the relationship between measured systemic concentration and molecular target exposure. Consequently, comparisons between human exposure and experimental activity should use target-specific potency or affinity together with unbound target-site exposure whenever these quantities are available.

For network pharmacology, this distinction is fundamental. A computationally plausible interaction may remain pharmacologically inaccessible if the relevant active molecular species does not reach the proposed target at a sufficient exposure level. Conversely, local gastrointestinal or tissue exposure may make selected mechanisms more plausible than would be inferred from plasma measurements alone. Target prioritisation should therefore incorporate measured human exposure, metabolite identity, tissue distribution where available, target-specific potency or affinity, and direct evidence of target engagement, rather than network position or docking affinity alone.

### 6.2. Tissue Exposure and Blood–Brain Barrier Considerations

Tissue exposure can differ substantially from circulating exposure. This distinction is particularly relevant to orally administered phytochemicals because gastrointestinal tissues may encounter the parent compound before extensive first-pass metabolism. Direct human measurements support this principle for curcumin, EGCG, and resveratrol [48,51,52].

For curcumin, oral administration of 3.6 g/day resulted in measurable parent compound in both normal and malignant colorectal tissue despite only trace peripheral exposure [48]. These observations support the plausibility of local gastrointestinal pharmacology even when systemic parent concentrations are low. However, colorectal tissue exposure should not be extrapolated to distant organs without corresponding tissue-specific measurements.

Likewise, EGCG and its methylated derivative have been detected directly in human prostate tissue following repeated green-tea consumption [51]. These data establish tissue access but do not demonstrate that unbound intracellular concentrations at a particular molecular target are sufficient to reproduce mechanisms identified in higher-concentration cellular experiments.

Resveratrol provides a further example of tissue-specific exposure. Parent resveratrol and multiple glucuronide and sulfate metabolites have been quantified in human colorectal tissue after repeated oral administration, with substantial variation according to anatomical location and individual patient [52]. Such findings emphasise that plasma exposure alone may not accurately describe local gastrointestinal exposure.

For neurological indications, the blood–brain barrier (BBB) introduces an additional requirement. Robust quantitative human brain target-site exposure data remain limited for curcumin, berberine, and EGCG in the current reference set. Systemic dosing, peripheral pharmacodynamic activity, or preclinical CNS effects should therefore not be interpreted as evidence of clinically relevant human brain target engagement.

For resveratrol, human evidence is somewhat stronger. In a randomised Phase II study in patients with Alzheimer’s disease, parent resveratrol and several metabolites were measurable in cerebrospinal fluid, demonstrating CNS access of resveratrol-derived molecular species [69]. Nevertheless, detection in cerebrospinal fluid does not establish adequate unbound concentration at a specific neural target, nor does it demonstrate target engagement or causal contribution to a clinical outcome.

Thus, tissue detection and BBB penetration are necessary but insufficient evidence for target-specific pharmacology. For CNS mechanisms in particular, translational confidence requires quantitative brain or CSF exposure where feasible, identification of the pharmacologically active molecular species, and corresponding pharmacodynamic or target-engagement evidence.

### 6.3. The Pharmacokinetic–Pharmacodynamic Disconnect

The divergence between experimental activity and achievable human exposure represents a major translational challenge for phytochemical pharmacology. This relationship cannot be assessed using plasma parent-compound concentration alone because local gastrointestinal exposure, tissue distribution, metabolite formation, formulation, and interindividual pharmacokinetic variability can substantially modify exposure [48,49,50,51,52,53,67,68].

These factors should not, however, be invoked automatically to explain an apparent exposure gap. Higher tissue concentration does not establish target engagement or therapeutic efficacy, and detection of a metabolite does not establish that the metabolite is pharmacologically active. Likewise, downstream modulation of a signalling pathway after dosing does not demonstrate that a predicted molecular target was directly engaged.

A stronger translational chain is:

administered dose → systemic and/or local exposure → active molecular species → target-site exposure → target engagement → proximal pharmacodynamic response → functional or clinical outcome.

Each step addresses a distinct pharmacological question. Pharmacokinetic measurements establish exposure; biochemical or biophysical studies establish the capacity for molecular interaction under defined conditions; target-engagement approaches determine whether the proposed interaction occurs in the relevant biological environment; and pharmacodynamic measurements assess whether the expected biological response accompanies molecular engagement [19,26]. Clinical benefit represents a further evidentiary step and should not be inferred from pharmacodynamic activity alone.

For network pharmacology, this distinction is fundamental because computational target prediction, PPI topology, molecular docking, and pathway enrichment can generate biologically plausible associations without establishing whether sufficient active compound reaches the proposed targets. Pharmacokinetic filtering should therefore be incorporated into target prioritisation before broad network convergence is interpreted as therapeutically relevant polypharmacology.

Where sufficient quantitative information is available, physiologically based pharmacokinetic (PBPK) modelling can integrate dose, absorption, metabolism, physiological parameters, and tissue distribution to estimate exposure at anatomical sites that are difficult to sample directly [70]. Such predictions remain dependent on model structure, parameter quality, assumptions, and validation and should therefore complement rather than replace direct measurements of tissue exposure, target engagement, and pharmacodynamic response [70].

For translational interpretation, proposed mechanisms can be considered as computationally plausible but pharmacokinetically unsupported, exposure-compatible but causally unresolved, or exposure-compatible and experimentally supported. These categories distinguish mechanisms lacking evidence of sufficient target-site exposure from those for which exposure is plausible but direct engagement or functional necessity remains uncertain, and from those supported by convergent evidence from exposure, target engagement, pharmacodynamics, function, and, where available, in vivo or clinical studies.

## 7. Challenges and Controversies

Despite the value of network pharmacology for organising complex phytochemical–target relationships, several methodological and pharmacological limitations constrain mechanistic interpretation. These include assay interference, incomplete target validation, publication and reporting bias, limitations of computational prediction, and variability in phytochemical composition. These limitations do not invalidate phytochemical research; rather, they define the level of evidence required before computational or pathway-level associations can be interpreted as pharmacologically meaningful polypharmacology.

### 7.1. Assay Interference, PAINS Liability, and Target Validation

Certain phytochemicals can exhibit substantial assay-interference liabilities, with curcumin as a prominent example due to its chemical reactivity, instability, aggregation-related behaviour, and potential interference across multiple assay formats [12,33,34]. Consequently, activity observed in a single biochemical or cellular assay should not, by itself, be interpreted as evidence of a specific molecular interaction.

PAINS alerts should be regarded as methodological warnings rather than evidence that a compound is biologically inactive [33,34]. Curcumin illustrates this distinction: its electrophilic and chemically reactive properties can generate nonspecific effects under some experimental conditions, while the same chemistry may also permit biologically relevant reactions with susceptible nucleophilic residues in proteins [12,41]. Thus, reported activity should neither be dismissed solely because of assay-interference liability nor accepted as target-specific without independent validation.

The distinction among chemical reactivity, biochemical interaction, cellular pathway modulation, physical target engagement, and causal target dependence is therefore essential. A biochemical interaction with an isolated protein provides evidence of molecular activity under defined conditions but does not establish cellular or physiological relevance. Conversely, loss of a phenotype following genetic perturbation supports functional involvement but does not independently demonstrate direct physical binding [19].

For compounds with substantial assay-interference liability, target validation should therefore rely on complementary and mechanistically independent approaches. These may include orthogonal biochemical assays, cellular target-engagement methods such as the cellular thermal shift assay (CETSA), chemoproteomic approaches, genetic perturbation, rescue or resistant-mutant experiments, and structure–activity analysis [19,26]. Confidence in a proposed molecular target is greatest when direct or orthogonal target-engagement evidence converges with functional perturbation and a phenotype compatible with pharmacologically achievable exposure.

Berberine illustrates the complementary principle that absence of a prominent assay-interference concern does not itself establish mechanistic validity. Clinical studies and meta-analyses support the effects of berberine on selected glycaemic and lipid outcomes, while multiple metabolic and microbiome-associated mechanisms have been proposed [14,60,71]. Thus, assay-interference liability and pharmacological validity are distinct questions; mechanistic validity additionally requires exposure compatibility, target engagement, functional contribution, and biological relevance.

### 7.2. Reproducibility and Publication Bias

Publication and selective-reporting bias are established concerns in biomedical research and can distort the apparent consistency of a literature by preferentially representing positive findings [72]. This issue is relevant to phytochemical pharmacology because repeatedly reported molecular or cellular associations may subsequently be incorporated into mechanistic reviews, databases, and computational target networks.

Reproducibility is also affected by the quality of the experimental system. Cell-line misidentification and contamination are documented sources of irreproducibility in biomedical research [73]. For chemically challenging phytochemicals, compound identity, purity, stability, and assay conditions can further influence experimental outcomes [12]. Botanical preparations introduce additional variability because their composition may depend on source material, cultivation, processing, extraction, purification, and storage [11].

A predominance of positive reports should therefore not be interpreted automatically as evidence of a large or reproducible biological effect. Publication selection, experimental heterogeneity, and assay-specific limitations may contribute to apparent consistency [34,72].

This issue is particularly important in network pharmacology because computational networks frequently incorporate previously reported compound–target relationships. Bias or error in the underlying literature and databases can therefore propagate into target networks and pathway-enrichment analyses [5]. Accordingly, network density, repeated database assignment, or high node connectivity should not be regarded as independent confirmation of pharmacological promiscuity or polypharmacology. Explicit evidence grading and independent experimental validation are required to reduce this risk.

### 7.3. Molecular Docking, Protein Flexibility, and Computational Overinterpretation

Molecular docking and related structure-based approaches are useful for generating and prioritising candidate phytochemical–target interactions, but computational scores are model-derived quantities rather than experimentally measured binding affinities or evidence of target engagement [19,25].

An important limitation is that proteins are conformationally dynamic. Docking against a single static structure may sample only one of several biologically relevant receptor states, and predicted ligand interactions can therefore depend on the selected structural conformation. Ensemble docking addresses this limitation by evaluating ligands across multiple receptor conformations and thereby incorporating receptor flexibility into structure-based prediction [25].

Nevertheless, greater computational sophistication does not convert a prediction into experimental evidence. A favourable docking score, a stable molecular-dynamics trajectory, or consistency across several computational models can strengthen the plausibility of a hypothesis, but none independently establishes direct binding, intracellular target engagement, or pharmacological relevance [19,25].

A stronger strategy is progressive orthogonal validation. Computationally prioritised interactions can be followed by biochemical interaction measurements, cellular target-engagement assays, and functional perturbation studies [19,26]. A relevant phytochemical example is provided by Leo et al., who combined docking and molecular-dynamics analysis of oleacein and oleocanthal with pharmacological inhibition of *PPARγ* and *PPARG* gene silencing [74]. Such convergence between computational prediction and functional perturbation substantially strengthens mechanistic attribution. However, pharmacological inhibition and gene silencing should still not be interpreted as direct biophysical evidence of ligand–receptor binding.

The same distinction applies to genetic validation more generally. Knockdown, knockout, or CRISPR dependency can demonstrate that a protein contributes functionally to a phenotype, but genetic dependency alone does not establish that a phytochemical physically binds that protein. Confidence in direct target attribution is strongest when functional perturbation converges with independent biochemical or cellular target-engagement evidence [19,26].

Large network-pharmacology target sets require similar caution. Predicted target lists can arise from chemical similarity, ligand-based inference, database mining, the literature-derived associations, or computational prediction and may substantially exceed the number of proteins actually engaged at pharmacologically relevant exposure [5,6]. PPI topology and pathway enrichment are likewise influenced by database coverage and annotation structure and should therefore be interpreted as prioritisation tools rather than direct measures of pharmacological importance [5].

Agreement among computational approaches can increase confidence in target prioritisation, but computational convergence remains distinct from experimental convergence. Docking, molecular dynamics, AI-based prediction, and network analysis are therefore best regarded as complementary hypothesis-generating tools rather than substitutes for biochemical interaction measurements, target engagement, functional perturbation, pharmacokinetic plausibility, or clinical evidence [19,24].

### 7.4. Standardisation and Phytochemical Characterisation

Standardisation represents an additional challenge in phytochemical research. Natural-product preparations can vary according to botanical source, cultivation and processing conditions, extraction, purification, storage, and formulation, resulting in differences in chemical composition and biological activity [11].

Curcuminoid preparations may contain differing proportions of curcumin and structurally related curcuminoids [13]. Green-tea preparations likewise contain mixtures of catechins whose abundance and stability can vary according to preparation and processing conditions [16,21]. Such compositional differences can influence pharmacokinetics, metabolism, assay behaviour, and interpretation of biological effects.

Standardisation is particularly important in network pharmacology because computational analyses depend on the identity of the compounds entered into the analytical model [5]. If an experimental preparation contains multiple constituents but the computational analysis represents only a single purified compound, the resulting target network may not accurately reflect the material experimentally tested. Conversely, the biological activity of a multicomponent preparation should not automatically be attributed to its most abundant constituent.

Mechanistic studies should therefore document the identity and composition of the tested material and provide appropriate chemical characterisation, particularly when extracts or multicomponent botanical preparations are investigated [11]. When network pharmacology is applied to such preparations, compounds included in the computational analysis should, where possible, correspond to chemically verified constituents of the material being tested.

Overall, the complexity of a computational network should not be equated with the complexity of pharmacological action. Confidence in a multi-target interpretation increases when chemical characterisation, computational prediction, biochemical interaction, cellular target engagement, functional validation, pharmacokinetic plausibility, and, where relevant, in vivo or clinical evidence are mutually consistent. This evidence chain helps distinguish credible polypharmacology from assay interference, database-derived association, downstream pathway convergence, and exposure-incompatible target predictions.

## 8. Clinical Evidence and Translational Status of Phytochemical Polypharmacology

Translation of network-derived mechanisms into clinically meaningful polypharmacology requires evidence that proposed molecular effects are achievable in humans and contribute to measurable pharmacodynamic or therapeutic outcomes. For this review, clinical efficacy refers to improvement in clinically meaningful outcomes. Pharmacodynamic evidence refers to measurable biological responses in humans that do not necessarily imply therapeutic benefit. Biomarkers are measurable biological indicators and are not assumed to predict clinical benefit. Intermediate endpoints occur along the pathway to a clinical outcome. In contrast, a surrogate endpoint is an intermediate measure used as a substitute for a clinically meaningful endpoint and requires appropriate validation for that purpose. Preclinical mechanistic evidence is considered separately because it cannot establish human therapeutic efficacy. Safety and tolerability are likewise distinguished from efficacy.

### 8.1. Oncology: Promise and Limitations

The clinical studies considered in this review do not establish stand-alone anticancer efficacy or clinically validated multi-target anticancer pharmacology for the representative phytochemicals discussed below.

In a Phase II study of patients with advanced pancreatic cancer, oral curcumin at 8 g/day was generally well tolerated and produced clinical and biological activity in a small number of patients, but evidence of objective antitumour efficacy was limited [66]. 25 patients were enrolled, and 21 were evaluable for response. The study also demonstrated low circulating curcumin exposure despite the high oral dose [66]. These findings support tolerability and limited human biological activity but do not establish curcumin as an effective anticancer therapy or demonstrate engagement of the multiple molecular targets proposed from preclinical studies.

A Phase IIa study of curcumin in colorectal neoplasia provides a different type of evidence. In this nonrandomized, open-label study, 44 eligible participants received 2 or 4 g/day curcumin for 30 days, with 41 completing treatment [75]. The 4 g/day dose was associated with a significant reduction in the number of aberrant crypt foci (ACFs), whereas the 2 g/day dose was not. However, the prespecified mucosal measures PGE_2_ and 5-HETE and the proliferation marker K_i_-67 were not significantly altered [75]. The reduction in ACF should therefore be interpreted as an intermediate finding and not as a validated surrogate for the prevention of colorectal cancer. Direct human tissue measurements from a separate study demonstrated measurable curcumin and metabolites in colorectal tissue after oral administration, supporting the plausibility of local gastrointestinal exposure despite low systemic parent-compound concentrations [48].

Green tea extract provides a complementary example. In a randomised Phase II study of patients with high-risk oral premalignant lesions, the overall clinical lesion-response rate was numerically higher with green-tea extract than with placebo but did not reach statistical significance [76]. Higher-dose treatment groups showed evidence of a dose–response relationship, whereas histological improvement was not statistically significant [76]. Because the intervention was a green tea extract rather than purified EGCG, these findings should not be attributed specifically to EGCG or interpreted as evidence that multiple proposed EGCG targets were engaged simultaneously. The results instead indicate a short-term lesion-response signal that requires confirmation in studies designed to evaluate cancer-preventive efficacy.

These oncology studies illustrate why extensive preclinical pathway modulation should not be equated with clinical polypharmacology. Demonstration of NF-κB, STAT3, Nrf2, apoptotic, or other signalling effects in experimental models does not establish that the corresponding molecular targets are simultaneously engaged at achievable human exposure. Clinical mechanistic attribution, therefore, requires an exposure-compatible link among molecular engagement, pharmacodynamic response, and a clinically meaningful outcome.

### 8.2. Metabolic Disease

Berberine has a comparatively substantial human evidence base for effects on metabolic biomarkers and intermediate endpoints. Meta-analyses of randomised studies have reported reductions in measures of glycaemia, including fasting and postprandial glucose and HbA1c, together with improvements in selected lipid parameters [71,77]. However, the earlier meta-analysis identified limitations in the methodological quality of the available studies [71]. The evidence, therefore, supports effects on selected metabolic endpoints but should not be interpreted as equivalent to definitive evidence of improvement in long-term cardiovascular, microvascular, or mortality outcomes.

Mechanistic studies implicate mitochondrial energy metabolism and downstream AMPK activation in the pharmacology of berberine [44]. In humans, the PREMOTE randomised clinical study demonstrated treatment-associated changes in gut microbial composition and bile acid metabolism and provided mechanistic evidence implicating altered *Ruminococcus bromii*-associated deoxycholic acid biotransformation in the metabolic response [57]. This evidence supports a microbiome-mediated contribution more strongly than microbial association alone. However, it does not establish that this mechanism accounts for all of the observed metabolic benefit, nor does it establish AMPK activation as the causal mechanism in humans.

Berberine, therefore, illustrates an important distinction between evidence of human metabolic activity and evidence of its molecular cause. Improvement in HbA1c, glucose, or lipid parameters does not demonstrate simultaneous engagement of multiple predicted targets or establish network-level polypharmacology. Prospective studies integrating parent-compound and metabolite exposure, mechanistically proximal pharmacodynamic biomarkers, and, where feasible, direct target-engagement measurements are needed to link clinical responses to specific molecular mechanisms.

### 8.3. Neurodegeneration

Clinical translation has been less convincing for curcumin and resveratrol in established Alzheimer’s disease.

In a randomised, double-blind, placebo-controlled study of 36 participants with mild-to-moderate Alzheimer’s disease, curcumin at 2 or 4 g/day did not produce significant differences in cognitive outcomes or the measured plasma and cerebrospinal fluid Alzheimer-related biomarkers compared with placebo [78]. Plasma concentrations of native curcumin were low. Although treatment was generally tolerated, several participants receiving curcumin withdrew because of gastrointestinal adverse effects [78]. Thus, the trial provided neither convincing clinical efficacy nor biomarker evidence of therapeutic activity under the conditions studied.

A randomised, double-blind, placebo-controlled Phase II study of resveratrol in 119 participants with mild-to-moderate Alzheimer’s disease demonstrated a different pattern [69]. Resveratrol and its major metabolites were measurable in plasma and cerebrospinal fluid, and treatment altered selected biomarker trajectories. The trial found no significant treatment effects on major cognitive and global measures, including ADAS-cog, MMSE, CDR-SOB, and NPI, although the decline in the secondary functional measure ADCS-ADL was smaller in the resveratrol group than in the placebo group [69]. Because the trial was primarily designed to evaluate safety, tolerability, pharmacokinetics, and biomarker outcomes, and was not powered to establish definitive clinical efficacy, this isolated secondary-outcome signal should be interpreted with caution. The investigators likewise emphasised that the observed biomarker changes did not establish clinical benefit [69].

These studies illustrate the distinction among CNS exposure, pharmacodynamic activity, intermediate biomarker responses, and clinical efficacy. Detection of a parent compound or metabolite in plasma or cerebrospinal fluid does not demonstrate that a specific neural target is engaged, and alteration of a biomarker does not necessarily translate into improvement in cognition, function, or disease progression.

For CNS indications, the exposure and target-accessibility considerations discussed in Section 6.2 are therefore central to interpretation. The stronger human CNS-exposure evidence available for resveratrol demonstrates access of resveratrol-derived molecular species to cerebrospinal fluid, but does not establish exposure-compatible engagement of any particular molecular target [69]. Corresponding quantitative human CNS target-site evidence remains substantially more limited for curcumin, berberine, and EGCG within the literature evaluated in this review.

The principal clinical studies discussed above, along with their study designs, major outcomes, human PK/PD or mechanistic information, and translational interpretations, are summarised in Table 4.

The studies summarised in Table 4 also illustrate why comparisons across diseases require attention to trial design, sample size, formulation and dose, exposure, endpoint type, and evidentiary purpose. A change in a biomarker, intermediate endpoint, lesion response, or metabolic measure should therefore be interpreted according to the clinical question the study was designed to answer, rather than being placed on the same evidentiary level as improvement in a validated clinical outcome.

### 8.4. Barriers to Clinical Translation

A recurrent translational problem is the disconnect between experimentally demonstrated biological activity and pharmacologically relevant human exposure. This constraint is well documented for curcumin, berberine, EGCG, and resveratrol [14,20,21,22]. As discussed in Section 6, local tissue exposure, metabolites, formulation, and interindividual variability can modify this relationship, but none should be invoked automatically to explain an apparent exposure mismatch. Improved plasma or tissue exposure likewise represents a pharmacokinetic observation and does not by itself establish molecular target engagement, pharmacodynamic benefit, or improvement in a clinically meaningful endpoint.

Clinical translation should therefore be interpreted in accordance with the exposure–target engagement–pharmacodynamic–outcome framework described in Section 6.3. Future clinical studies should incorporate mechanistically informative exposure and response measurements wherever feasible. Pharmacokinetic analyses should distinguish parent compounds from relevant metabolites; pharmacodynamic analyses should prioritise validated target- or pathway-proximal biomarkers; and tissue sampling, molecular imaging, or other target-accessibility measurements should be incorporated when scientifically justified and feasible. Trial design should additionally prespecify clinically meaningful primary outcomes, distinguish exploratory biomarkers and secondary endpoints from efficacy endpoints, and account appropriately for formulation, dose, disease stage, sample size, multiplicity, and statistical power.

The available clinical evidence supports different levels of human biological and pharmacological activity rather than a uniform demonstration of clinically meaningful polypharmacology. Berberine has comparatively stronger evidence of improvements in selected glycaemic and lipid endpoints, although the molecular mechanisms underlying these effects remain incompletely elucidated in humans [57,71,77]. Curcumin shows limited biological activity in advanced pancreatic cancer and an intermediate colorectal neoplasia signal, without established anticancer efficacy [66,75]. Green tea extract has shown a short-term lesion-response signal in oral premalignant lesions, without establishing cancer-preventive efficacy or EGCG-specific polypharmacology [76]. In Alzheimer’s disease, curcumin did not demonstrate convincing clinical or biomarker efficacy in the cited trial. In contrast, resveratrol showed CNS exposure and selected biomarker effects, along with an isolated secondary functional signal, but no consistent evidence of cognitive or global clinical benefit [69,78].

Overall, current clinical evidence does not establish that the therapeutic effects of these phytochemicals result from simultaneous engagement of the broad target networks proposed by computational or preclinical studies. More definitive mechanistic attribution will require the prospective integration of human exposure, target engagement, mechanistically proximal pharmacodynamic evidence, causal validation, and appropriately powered clinical outcome data.

## 9. Advanced Delivery Systems for Pharmacokinetic Optimisation

Poor aqueous solubility, limited absorption, rapid metabolism, chemical instability, and unfavourable tissue exposure can substantially constrain the translation of phytochemical pharmacology. Advanced formulation strategies are therefore being developed to improve physicochemical stability, dissolution, gastrointestinal absorption, systemic exposure, and tissue delivery [79].

Using the translational framework defined in Section 6.3, delivery systems are evaluated according to the furthest stage experimentally demonstrated, from formulation-induced pharmacokinetic improvement through tissue exposure and, where available, target engagement, pharmacodynamic response, and clinical outcome. Improved systemic or tissue exposure should therefore be regarded as enabling pharmacokinetic evidence rather than proof of molecular target engagement or therapeutic efficacy.

### 9.1. Phytosomes

Phytosomes are phospholipid-based complexes developed to improve the absorption of poorly bioavailable phytochemicals. For curcumin, the lecithin-based formulation Meriva^®^ has been evaluated directly in humans [23].

In a randomised crossover pharmacokinetic study, total curcuminoid absorption was approximately 29-fold greater with Meriva than with the corresponding unformulated curcuminoid mixture [23]. Importantly, this increase represented total curcuminoid absorption rather than a 29-fold increase in circulating unchanged parent curcumin. Only phase II metabolites were detectable in plasma, and the formulation altered the relative circulating profile of curcuminoid-derived species [23].

This study, therefore, demonstrates improvement at the formulation-to-pharmacokinetic level but does not demonstrate increased engagement of specific molecular targets. Improved absorption should not automatically be interpreted as evidence that the additional exposure reaches a relevant tissue in a pharmacologically active form, engages predicted targets, produces the expected pharmacodynamic response, or improves a clinically meaningful outcome.

Phytosome technology, therefore, illustrates an important translational distinction: formulation can reduce an exposure barrier without establishing the subsequent mechanistic sequence from tissue exposure to target engagement and therapeutic benefit.

### 9.2. Polymeric Nanoparticles

Polymeric nanoparticle formulations can protect chemically unstable compounds, modify drug release, and improve oral or tissue exposure under experimental conditions [80,81]. Curcumin nanoformulations have been extensively investigated as strategies to improve delivery and pharmacological performance [80].

For EGCG, a pH-sensitive polymeric nanoparticle formulation increased oral bioavailability by more than 2.4-fold relative to EGCG powder in a rat model and was associated with reduced proteinuria and improved renal histopathological scores in nephrotic-syndrome rats [81]. These findings provide preclinical formulation, pharmacokinetic, and disease-related phenotypic evidence, but they do not establish equivalent benefit in humans, direct exposure of a defined molecular target, or target engagement.

Thus, increased nanoparticle-associated systemic exposure, tissue accumulation, or intracellular uptake should primarily be interpreted as evidence of delivery or pharmacokinetics unless accompanied by direct target-engagement measurements and mechanistically proximal pharmacodynamic validation.

Polymeric nanocarriers also face translational challenges related to formulation reproducibility, physical and chemical stability, scale-up, manufacturing control, biodistribution, safety, and regulatory translation [79,80]. These issues become particularly important when therapeutic performance depends on reproducible particle characteristics and tissue-specific disposition.

### 9.3. Self-Microemulsifying Drug Delivery Systems

Self-microemulsifying drug delivery systems (SMEDDS) are designed to improve the dissolution and gastrointestinal absorption of poorly water-soluble compounds. A curcumin-loaded SMEDDS markedly increased curcumin solubility and dissolution and enhanced oral absorption relative to a curcumin suspension in preclinical studies [82].

These findings establish formulation and preclinical pharmacokinetic improvements, but they do not demonstrate that the increased exposure results in greater molecular target engagement, a corresponding pharmacodynamic response, or clinical efficacy. For network-derived mechanisms, the critical next question is whether formulation-induced increases in exposure translate into exposure of the relevant active molecular species at the proposed tissue and intracellular targets.

Accordingly, SMEDDS should presently be regarded as an exposure-enhancing delivery strategy, whose mechanistic and clinical significance remain dependent on downstream tissue distribution, target engagement, pharmacodynamic, safety, and efficacy studies.

### 9.4. Plant-Derived Extracellular Vesicle-like and Biomimetic Delivery Systems

Plant-derived extracellular vesicle-like nanoparticles are described using several partially overlapping terms in the literature, including plant-derived exosome-like nanoparticles and plant-derived extracellular vesicles [83,84]. These membrane-associated nanoscale structures can contain lipids, proteins, nucleic acids, and other molecular cargo and are increasingly being investigated as biogenic delivery platforms because of their potential for cellular uptake, cargo protection, and tissue delivery [83,84].

Their application to central nervous system delivery has attracted particular interest. Reviews of plant-derived vesicle-like systems describe their potential for blood–brain barrier transport and CNS drug delivery, although the supporting evidence remains predominantly preclinical [83].

More directly, a recent preclinical study evaluated plant-derived extracellular vesicles loaded with itraconazole using glioblastoma-cell assays and an in vitro blood–brain barrier permeability model [85]. Ginger- and aloe-derived vesicles demonstrated permeability in that experimental system. Because itraconazole is not itself a phytochemical and the BBB evidence was obtained using an in vitro model, this study supports the feasibility of the plant-derived vesicle carrier platform rather than demonstrating CNS delivery of a phytochemical, in vivo brain exposure, or molecular target engagement [85].

These findings should therefore remain classified as predominantly preclinical evidence of delivery. Demonstration of cellular uptake or barrier permeability does not establish that an encapsulated compound reaches an intracellular molecular target at an exposure sufficient to produce a pharmacological effect. In CNS applications, BBB passage, brain exposure, target engagement, pharmacodynamic response, and therapeutic efficacy must be regarded as separate evidentiary stages.

Important translational challenges include standardisation of isolation and characterisation procedures, particle heterogeneity, reproducibility, cargo definition, stability, scalable production, biodistribution, immunological effects, and long-term safety [83,84]. Comparative superiority over established delivery systems, therefore, requires direct evaluation of pharmacokinetic, pharmacodynamic, safety, and efficacy rather than inference from cellular uptake or barrier-crossing capability alone.

### 9.5. Comparative Assessment

Within the evidence evaluated in this review, none of the delivery platforms discussed above has been shown to complete the full progression from formulation optimisation to a clinically meaningful outcome for phytochemical polypharmacology. Human pharmacokinetic evidence is available for the curcumin phospholipid formulation Meriva^®^, which substantially increased total curcuminoid absorption relative to an unformulated preparation [23]. By contrast, the polymeric EGCG nanoparticle and curcumin SMEDDS studies included in the present bibliography provide primarily preclinical evidence for improved oral exposure and related biological effects [81,82]. At the same time, plant-derived extracellular vesicle-like systems remain emerging and predominantly preclinical approaches [83,84,85].

These platforms should therefore not be compared solely according to fold increases in apparent bioavailability, intracellular uptake, or theoretical targeting potential. Their interpretation should instead reflect the furthest experimentally demonstrated stage of translation and consider biodistribution, metabolite exposure, selectivity, and tolerability, particularly because increased exposure may also increase off-target pharmacology or safety liabilities [79].

From a network-pharmacology perspective, advanced delivery systems are best regarded as exposure-modifying technologies rather than evidence of a polypharmacological mechanism. Their translational value depends on whether improved exposure ultimately supports pharmacologically relevant tissue concentrations, target engagement, appropriate pharmacodynamic activity, acceptable safety, and clinically meaningful benefit.

## 10. Next-Generation Technologies Enabling Predictive Phytochemical Pharmacology

Next-generation computational and experimental technologies are expanding the ability to integrate chemical, structural, genetic, transcriptomic, spatial, and pharmacokinetic information in drug-discovery workflows. Their principal value in phytochemical research is to improve hypothesis prioritisation, biological resolution, and experimental testing rather than to establish a mechanism independently [19,86]. Consistent with the evidence framework developed in Section 3.2, computational hypotheses should therefore be progressively evaluated using biochemical evidence, cellular target engagement, functional perturbation, pharmacokinetic/pharmacodynamic assessment, and, where appropriate, in vivo and clinical evidence.

### 10.1. Artificial Intelligence-Driven Network Pharmacology

Artificial intelligence and machine-learning approaches are increasingly being applied to drug–target prediction and systems-level target prioritisation [86,87]. Different approaches use distinct molecular representations. DeepDTA encodes ligand SMILES strings and protein amino-acid sequences using convolutional neural networks to predict drug–target binding affinity, whereas GraphDTA introduced graph-based molecular representations to capture ligand topology [88,89]. Predicted affinities from either approach remain computational estimates rather than experimentally measured binding affinities or evidence of cellular target engagement.

Graph neural networks, multimodal approaches, and knowledge graphs can further integrate molecular and biological information for target identification or drug repurposing applications [86,87,90]. Such integration can improve candidate prioritisation, but prediction quality remains dependent on the underlying data, molecular representation, model assumptions, and biological coverage [24,86].

Particular caution is required when interpreting model performance mechanistically. Training–test overlap, biased datasets, limited applicability domains, and other validation problems can lead to apparently strong predictive performance that may not generalise prospectively [24]. AI predictions should therefore be evaluated using appropriately separated validation data and, ultimately, prospective experimental testing. Model explanations or feature-attribution methods may help identify factors contributing to a prediction, but they do not establish biological causality.

In phytochemical pharmacology, AI-derived targets are therefore best regarded as candidates for prioritisation. Their translational relevance should subsequently be assessed in relation to compound identity, structural plausibility, pharmacologically achievable exposure, existing experimental evidence, and the feasibility of direct validation.

### 10.2. Structural Biology and CRISPR-Based Functional Validation

Predicted protein structures provide an additional resource for evaluating computationally proposed compound–protein interactions. The AlphaFold Protein Structure Database substantially expands access to predicted protein structures, including proteins for which experimentally determined structures may be unavailable [91]. Such structures can support computational hypothesis generation, but they do not demonstrate that a phytochemical binds the predicted protein.

Structure-based predictions are also sensitive to protein conformation. Proteins are dynamic rather than rigid structures, and docking against a single conformation may not represent the full range of ligand-accessible states. Ensemble docking provides one strategy for incorporating receptor flexibility by evaluating ligands across multiple protein conformations [25]. Accordingly, a favourable docking pose or a stable molecular dynamics trajectory should be interpreted as increased structural plausibility rather than as proof of molecular binding or target engagement, and should be strengthened by orthogonal experimental evidence [19].

CRISPR-based genetic screening and perturbation provide complementary approaches for identifying genes that influence cellular phenotypes or drug responses [30,31]. However, a gene identified through a CRISPR screen should not automatically be classified as the direct molecular target of a phytochemical. Genetic dependency may arise through upstream or downstream signalling, transport, metabolism, stress responses, or other cellular processes [31].

Genetic dependency and physical target engagement, therefore, address different mechanistic questions. Confidence in direct target attribution increases when genetic evidence converges with biochemical or biophysical interaction data and cellular target-engagement measurements [19,31]. CETSA, for example, assesses compound-induced changes in protein thermal stability in cells or tissues and can provide evidence of target engagement in a biological environment [26]. Rescue experiments, resistant mutants, structure–activity relationships, and complementary chemical-biology approaches can provide additional functional support where appropriate [19].

These approaches provide complementary forms of evidence within the hierarchy defined in Section 3.2: biochemical and biophysical studies assess molecular interactions; target-engagement methods assess interactions in biological systems; and genetic perturbation evaluates functional dependence. Genetic dependency should therefore not be considered equivalent to direct molecular binding.

### 10.3. Single-Cell Transcriptomics and Spatial Omics

Single-cell RNA sequencing enables transcriptome profiling at cellular resolution and can resolve molecular heterogeneity that is obscured in bulk-tissue measurements [92,93]. When applied to pharmacological experiments, this resolution can be used to identify cell populations exhibiting distinct transcriptional responses to treatment.

Spatial transcriptomics extends molecular profiling by retaining anatomical information within tissues, while newer spatial multi-omics approaches can integrate multiple molecular layers and characterise spatially organised biological states [94,95,96]. These technologies can therefore help determine where and in which cellular populations treatment-associated molecular responses occur.

Their outputs should not, however, be interpreted as direct evidence of target engagement. Changes in transcript abundance, pathway scores, or spatial molecular patterns are generally downstream observations and do not independently identify the molecular event responsible for the response. Likewise, computationally inferred interactions derived from expression data remain hypotheses until experimentally tested.

The principal contribution of single-cell and spatial technologies to phytochemical pharmacology is therefore improved biological and anatomical resolution. Candidate mechanisms identified through these approaches can subsequently be examined using cell-type-specific perturbation, protein-level measurements, biochemical validation, or target-engagement approaches. Thus, single-cell and spatial analyses can refine mechanistic hypotheses but do not independently establish direct molecular engagement or therapeutic efficacy.

### 10.4. PBPK Modelling and Quantitative Systems Integration

Physiologically based pharmacokinetic (PBPK) modelling provides a quantitative framework for integrating compound-specific pharmacokinetic properties with physiological parameters to predict systemic and tissue exposure. Its application to dietary phytochemicals is particularly relevant because experimentally proposed mechanisms may occur at concentrations that differ substantially from achievable systemic or tissue exposure [70].

PBPK modelling can complement network pharmacology by testing whether predicted compound–target relationships are pharmacokinetically plausible. Where sufficient data are available, candidate mechanisms can be evaluated in relation to dose, absorption, metabolism, tissue distribution, metabolite formation, and estimated exposure at relevant anatomical sites [70].

However, model predictions are dependent on the quality and completeness of the underlying physiological and compound-specific parameters. Uncertainty in absorption, metabolism, protein binding, tissue partitioning, or other inputs can propagate into predicted exposure. PBPK predictions should therefore be compared with measured pharmacokinetic data whenever possible and should complement rather than replace experimental measurements [70].

Integration of exposure modelling with network, structural, and omics information may ultimately help prioritise mechanisms that are both biologically plausible and compatible with achievable exposure. At present, however, the appropriate objective is not automated prediction of therapeutic efficacy but quantitative assessment of whether a proposed mechanism is pharmacokinetically plausible and experimentally testable.

The complementary roles, principal limitations, recommended validation strategies, and evidentiary positions of these computational, experimental, pharmacokinetic, and clinical approaches are summarised in Table 5.

Collectively, these technologies should be regarded as complementary components of an evidence-building framework rather than interchangeable forms of mechanistic validation. AI and network approaches prioritise hypotheses; structural methods refine molecular plausibility; single-cell and spatial technologies improve cellular and anatomical resolution; genetic perturbation strengthens functional attribution; target-engagement approaches test molecular interaction in biological systems; and PBPK modelling imposes quantitative exposure constraints [19,26,70,86].

The translational objective is to connect these approaches into an evidence chain extending from chemical identity and computational prediction to molecular engagement, functional contribution, exposure compatibility, pharmacodynamic response, and clinical outcome. Such integration may reduce unsupported target assignments and improve discrimination between credible, exposure-compatible polypharmacology and computationally inferred pathway convergence.

## 11. Future Directions: Toward Exposure-Aware and Precision Phytopharmacology

Future phytochemical pharmacology should increasingly shift from broad expansion of predicted target networks toward mechanisms that are experimentally testable, pharmacologically accessible, and relevant to human exposure. Integrating computational prioritisation with quantitative pharmacokinetics, tissue exposure, mechanistic validation, pharmacodynamic assessment, and appropriately designed clinical investigation should enable more selective evaluation of candidate mechanisms while reducing overinterpretation of network-level associations.

### 11.1. Semi-Synthetic Derivatives and Multi-Target Medicinal Chemistry

Medicinal chemistry optimisation offers a potential strategy for addressing chemical and pharmacokinetic limitations of natural products while retaining desirable pharmacological properties [11]. Curcumin provides a relevant example because its chemical instability, reactivity, and pharmacokinetic limitations have stimulated extensive investigation of structural analogues and modified derivatives [12].

For berberine, structural modification can likewise alter pharmacokinetic and pharmacological behaviour. Dihydroberberine, for example, has been investigated as a more bioavailable berberine derivative and retains effects on mitochondrial complex I and downstream AMPK-associated signalling [44]. Such findings illustrate how chemical modification can alter exposure while preserving selected elements of the parent compound’s pharmacology.

More broadly, natural-product scaffolds provide starting points for medicinal-chemistry optimisation, while designed multiple-ligand strategies provide a conceptual framework for engineering activity across more than one therapeutically relevant target [2,8,11]. The objective should not, however, be to maximise biochemical potency or preserve the largest possible number of predicted targets. A more potent derivative may remain translationally limited if metabolic stability, tissue exposure, or target accessibility is inadequate, whereas reducing chemically reactive or therapeutically irrelevant interactions may improve pharmacological selectivity.

Network pharmacology may assist this process by prioritising disease-associated targets and molecular modules for experimental evaluation [1,5]. A more rigorous strategy would combine computational prioritisation with structure–activity relationships, orthogonal biochemical assays, cellular target engagement, functional perturbation, and PK/PD assessment. The goal is therefore evidence-guided multi-target optimisation rather than preservation of broad molecular promiscuity.

### 11.2. Biomarker-Guided and Exposure-Aware Precision Phytotherapy

Inter-individual pharmacokinetic and biological variability may contribute to heterogeneous responses to phytochemicals. Human studies of berberine, for example, demonstrate substantial inter-individual pharmacokinetic variability, while both clinical and mechanistic studies indicate that gut-microbial composition and metabolism can alter berberine disposition and metabolic responses [45,57,68].

Biological variability alone, however, does not establish a clinically useful predictive biomarker. Future studies should therefore move beyond descriptive molecular signatures toward prospectively validated biomarkers linked to exposure, pharmacodynamic response, and clinically meaningful outcomes. Candidate biomarkers may include metabolite profiles, pathway-associated molecular signatures, genetic features, microbiome-derived variables, or tissue-specific molecular patterns, but their predictive value should be assessed prospectively and independently.

The microbiome is particularly relevant because microbial metabolism can alter phytochemical disposition and generate molecular species with absorption or biological properties that differ from those of the administered parent compound [45,58]. Associations between microbial taxa and treatment response should therefore be distinguished from causal mediation. Stronger evidence can be generated using longitudinal metabolite measurements together with experimental approaches such as gnotobiotic models, defined microbial communities, microbial-transfer experiments, or targeted manipulation of candidate organisms [58].

Precision approaches should also distinguish total systemic concentration from pharmacologically relevant target-site exposure. Tissue distribution, intracellular disposition, metabolite formation, protein binding, and target accessibility can all modify the relationship between administered dose and molecular response. Accordingly, a biomarker-defined subgroup should not be considered pharmacologically responsive solely because it expresses a pathway-associated signature; stronger evidence would demonstrate that the biomarker predicts exposure-compatible target engagement, a proximal pharmacodynamic response, or a clinically meaningful outcome.

### 11.3. Systems-Guided and Exposure-Aware Target Prioritisation

The future role of network pharmacology should increasingly shift from broad target enumeration to the integrated prioritisation of exposure-compatible and causally testable targets or combinations thereof. AI and multi-omics approaches can contribute molecular and disease-context information, while PBPK modelling can impose quantitative constraints by estimating whether relevant exposure is achievable within specific physiological compartments [70,86,87].

These approaches should be regarded as components of an evidence-building framework rather than automated mechanism-discovery systems. Network enrichment and topology are influenced by database and annotation coverage, whereas AI predictions depend on training data, model assumptions, applicability domains, and validation strategy [5,24]. Pharmacokinetic constraints should therefore be incorporated during target prioritisation by considering systemic and tissue exposure, metabolite formation, intracellular accessibility, target abundance, biochemical potency or affinity, exposure duration, and the relationship between exposure and pharmacodynamic response [70].

Consistent with the translational categories defined in Section 6.3, targets that are computationally plausible but incompatible with available exposure data should receive lower translational priority unless local exposure or active metabolites provide quantitative support. Conversely, convergence of exposure compatibility with molecular, functional, or target-engagement evidence should increase confidence in a proposed mechanism. A smaller network of experimentally supported, exposure-compatible, and causally relevant targets may therefore provide greater translational information than a larger network dominated by computational associations. Candidate mechanisms can subsequently be advanced through the integrated validation framework summarised in Figure 2.

When combinations of compounds or targets are investigated, additive, synergistic, antagonistic, and compensatory effects should be experimentally distinguished. Formal interaction frameworks such as Bliss independence or Loewe additivity should be used where appropriate, rather than inferring synergy from pathway convergence alone [27].

### 11.4. Prospective Validation and Evidence-Tiered Clinical Development

Clinical development should remain proportional to the level of mechanistic evidence available. Early-phase studies need not establish every component of a proposed molecular mechanism, but hypotheses carried into clinical development should, where feasible, be linked to measurable exposure and pharmacodynamic endpoints. Formulation, dose, parent-compound and metabolite exposure, tissue accessibility, biomarker status, and clinical outcomes should be evaluated together according to the translational framework described in Section 6.3.

Similarly, computationally prioritised targets should not be promoted to clinical biomarkers solely based on network centrality, docking scores, AI confidence, pathway enrichment, or altered gene expression. Target attribution is strengthened when evidence of biochemical or cellular engagement converges with functional perturbation [5,19,26]. Future clinical studies should therefore use evidence-tiered designs in which mechanistic claims remain proportional to supporting evidence and predefined pharmacological hypotheses are evaluated alongside clinically meaningful endpoints.

### 11.5. Integrative Perspective

The principal opportunity for next-generation phytochemical pharmacology is not to generate increasingly complex interaction networks, but to determine which proposed mechanisms are experimentally supported, pharmacologically accessible, causally relevant, and therapeutically meaningful.

AI and network methods can prioritise hypotheses [86]; structural and biochemical approaches can refine molecular plausibility; single-cell and spatial technologies can improve cellular and anatomical resolution [95]; genetic perturbation can strengthen functional attribution; target-engagement approaches can test molecular interaction in biological systems [19,26]; advanced delivery systems can modify exposure; and PBPK modelling can assess whether proposed mechanisms are compatible with achievable systemic or tissue concentrations [70]. These approaches are complementary and should be interpreted according to the evidentiary questions each can address.

Future progress, therefore, requires a transition from network-rich descriptions to evidence-rich mechanisms. Precision phytopharmacology should not be defined by the number of targets assigned to a compound, but by the identification of targets or target combinations that are pharmacologically accessible and supported by convergent molecular, functional, pharmacokinetic, pharmacodynamic, and clinical evidence. Such an exposure-aware and evidence-graded strategy provides a more rigorous basis for translating systems-level phytochemical pharmacology into reproducible and clinically relevant mechanisms.

## 12. Conclusions

Network pharmacology provides a valuable framework for organising and prioritising potential multi-target mechanisms of phytochemicals, but its predictions should be distinguished from experimentally demonstrated pharmacology. Across curcumin, berberine, epigallocatechin-3-gallate (EGCG), and resveratrol, the available evidence supports varying levels of biochemical interactions, modulation of cellular pathways, functional activity, pharmacokinetic exposure, and human pharmacological effects. However, the current evidence does not establish that the clinical effects of these compounds result from the simultaneous engagement of the broad molecular networks frequently proposed in computational or preclinical studies.

The principal translational priority is therefore to identify mechanisms that are both experimentally supported and compatible with achievable target-site exposure. Clinically meaningful phytochemical polypharmacology will require convergent evidence linking molecular engagement with functional contribution, pharmacodynamic response, and appropriately designed human studies. In this context, a smaller set of well-supported and exposure-compatible targets may provide greater translational value than a large network dominated by computational predictions or downstream pathway associations.

## Figures and Tables

**Figure 1:**
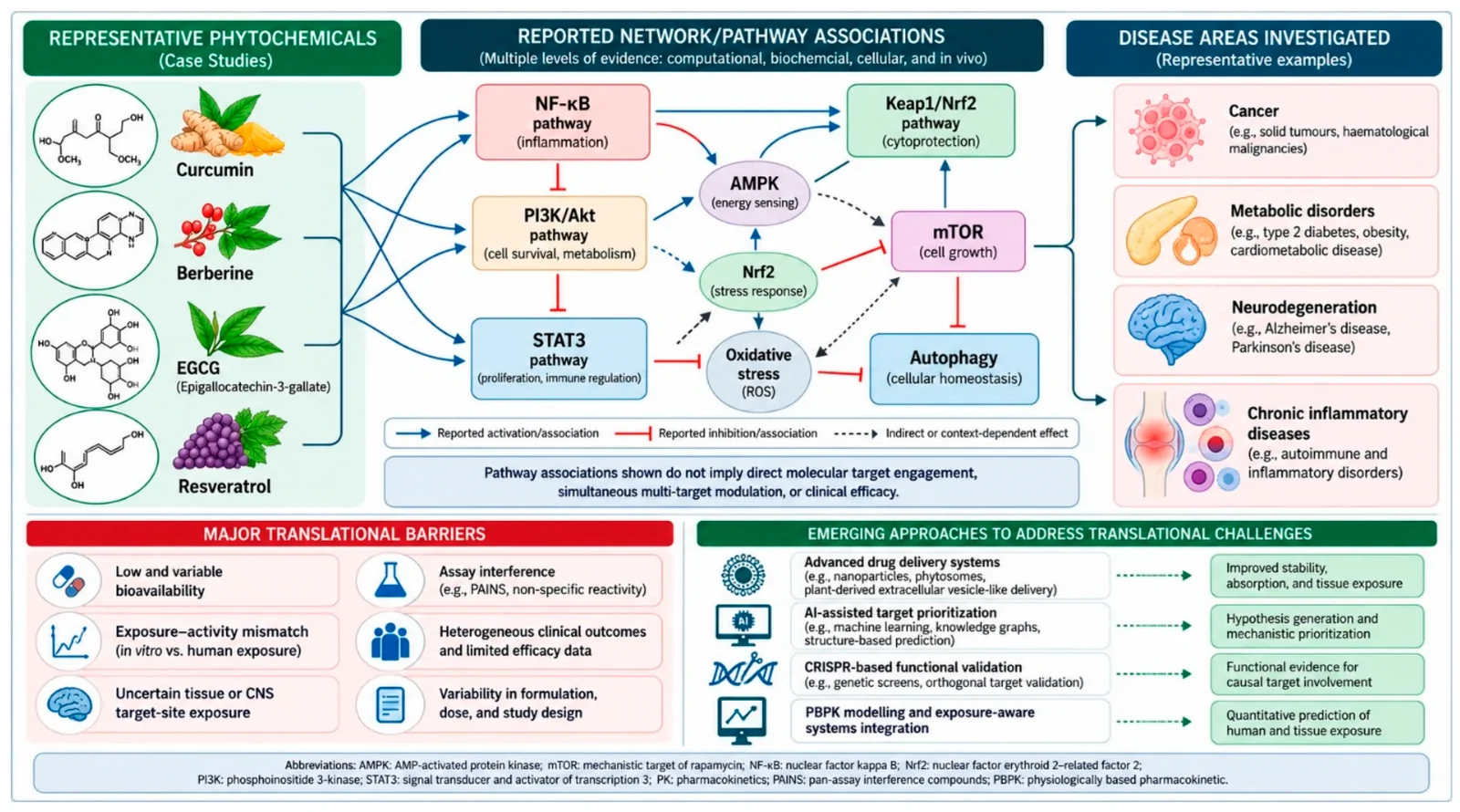
Conceptual framework for evidence-based evaluation of network pharmacology-guided phytochemical polypharmacology. Curcumin, berberine, epigallocatechin-3-gallate (EGCG), and resveratrol are representative phytochemicals associated with the modulation of interconnected inflammatory, metabolic, stress-response, and growth-regulatory pathways, including NF-κB, PI3K/Akt, AMPK/mTOR, STAT3, Nrf2/Keap1, oxidative stress, and autophagy-related networks. The illustrated pathway relationships represent heterogeneous levels of evidence and should not be interpreted as equivalent direct molecular target engagement, simultaneous multi-target modulation, pharmacological synergy, or clinical efficacy. Representative disease contexts include cancer, metabolic disorders, neurodegeneration, and chronic inflammatory diseases. Major translational barriers include low or variable bioavailability, exposure–activity mismatch, uncertain tissue or CNS target-site exposure, assay interference, heterogeneous clinical outcomes, and variability in formulation and study design. Emerging strategies include advanced drug-delivery systems, AI-assisted target prioritisation, CRISPR-based functional validation, and physiologically based pharmacokinetic (PBPK) modelling with exposure-aware systems integration. These approaches are intended to strengthen hypothesis prioritisation and translational assessment but require experimental confirmation of target engagement, pharmacodynamic response, and clinical relevance.

**Figure 2:**
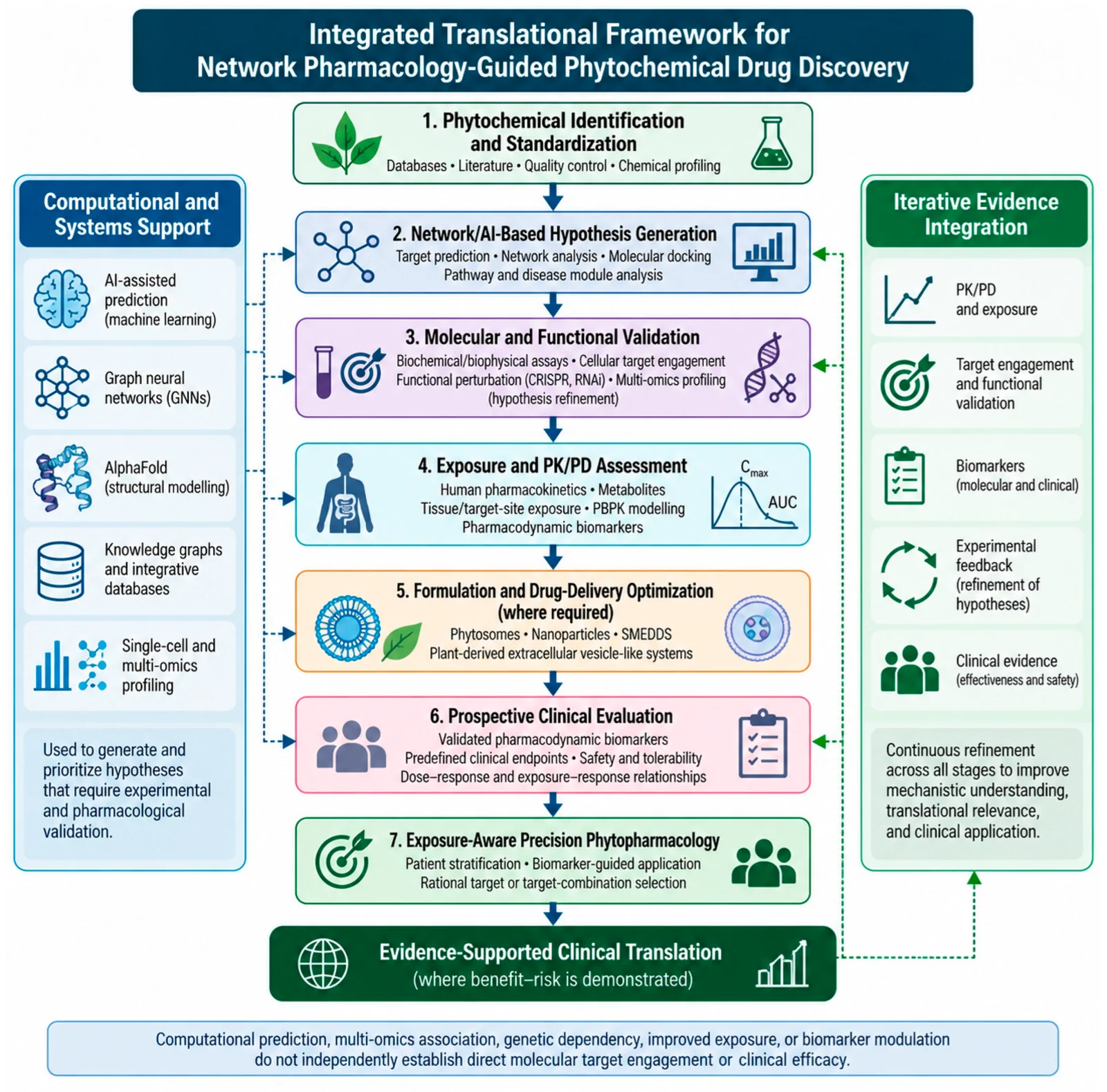
Integrated translational framework for evidence-based network pharmacology-guided phytochemical drug discovery. The framework depicts a staged and iterative workflow from phytochemical identification and standardisation through network- and AI-assisted hypothesis generation, molecular and functional validation, exposure and PK/PD assessment, formulation optimisation where required, prospective clinical evaluation, and exposure-aware precision phytopharmacology. Computational and systems approaches—including AI-assisted prediction, graph neural networks, AlphaFold-based structural modelling, knowledge graphs, and single-cell/multi-omics profiling—support hypothesis generation and refinement, whereas biochemical and biophysical assays, cellular target-engagement methods, genetic perturbation, human pharmacokinetics, tissue-exposure measurements, PBPK modelling, pharmacodynamic biomarkers, and clinical evidence provide complementary levels of validation. Iterative evidence integration across these stages is required because computational prediction, multi-omics association, genetic dependency, increased exposure, or biomarker modulation alone does not establish direct molecular target engagement or clinical efficacy.

**Table 1:** Compound-Centred Translational Summary of Representative Phytochemicals.

Phytochemical	Chemical Class	Direct or Putative Molecular Interactions	Major Downstream Signalling	Exposure/PK Consideration	Principal Translational Limitation
Curcumin	Diarylheptanoid	Electrophilic/cysteine-reactive chemistry and multiple reported protein interactions; covalent interactions with proteins, including thioredoxin reductase and IKKβ, have been reported in biochemical systems, but their contribution at clinically relevant exposure remains uncertain [12,41]	NF-κB, STAT3, PI3K/Akt and related signalling; evidence is predominantly cellular/preclinical and does not by itself demonstrate direct target engagement [42,43]	Human systemic exposure to unconjugated parent curcumin is generally very low after conventional oral dosing; even enhanced formulations often yield plasma concentrations below those used in many mechanistic in vitro studies [20,35]. Local gastrointestinal tissue exposure may nevertheless exceed plasma exposure; curcumin and conjugated metabolites have been detected in human colorectal tissue after high oral doses [48]	Low systemic parent exposure, rapid metabolism, chemical instability, electrophilic reactivity and assay interference; the relevance of many reported molecular targets to human exposure therefore remains uncertain [12,20]
Berberine	Isoquinoline alkaloid	Functional inhibition of mitochondrial respiratory complex I has been demonstrated experimentally, with downstream AMPK activation; the study did not establish binding to a defined complex-I molecular subunit [44]	AMPK-associated metabolic signalling and related downstream pathways; these pathway effects should not be interpreted as evidence that all pathway components are directly engaged [44]	Human circulating parent-berberine concentrations are very low after oral administration. Intestinal first-pass effects, tissue distribution, host metabolism, and microbiota-dependent conversion to dihydroberberine contribute to its complex pharmacokinetic profile [14,45,49]	Low systemic parent exposure, together with potentially important intestinal, tissue, metabolic and microbiota-dependent mechanisms, complicates extrapolation from plasma exposure to pharmacological activity
EGCG	Catechin polyphenol	Direct binding to the 67 kDa laminin receptor (67LR) has been demonstrated by surface plasmon resonance with nanomolar affinity [46]; EGCG also inhibited DNMT activity in nuclear-extract assays (Ki ≈ 6.9 μM); however, selective direct engagement of purified DNMT1 was not established in that study [17]	PI3K/Akt, MAPK, NF-κB, Nrf2 and receptor-associated signalling; much of the evidence is cellular/preclinical rather than direct molecular target-engagement evidence [7,21]	Oral EGCG produces measurable but limited systemic exposure, with dose-dependent pharmacokinetics in humans [50]. EGCG can also be detected in human tissues—for example, prostate tissue after green-tea intake—but unbound intracellular and target-site concentrations relevant to many proposed mechanisms remain incompletely characterised [16,21,50,51]	Chemical instability and auto-oxidation, limited and variable systemic exposure, and incomplete demonstration that many proposed molecular interactions occur at relevant unbound tissue concentrations
Resveratrol	Stilbene polyphenol	Direct SIRT1 activation remains disputed and appears assay/substrate dependent [18]; Inhibition of cAMP phosphodiesterases has been reported as an alternative upstream mechanism leading indirectly to AMPK- and SIRT1-associated signalling [47]	cAMP–Ca^2+^–CaMKKβ–AMPK–NAD^+^/SIRT1-associated signalling, together with broader metabolic, inflammatory and stress-response effects; evidence varies from biochemical and cellular studies to animal models [7,47]	Oral resveratrol is substantially absorbed but undergoes extensive glucuronidation and sulfation, producing low circulating concentrations of the unchanged parent compound after conventional dosing [22]; Parent resveratrol and metabolites can nevertheless reach substantially higher local concentrations in gastrointestinal tissues, and systemic exposure is formulation-dependent [52,53]	Extensive metabolism, substantial formulation- and tissue-dependent differences in exposure, and continuing uncertainty regarding historically proposed direct targets such as SIRT1

Interpretive note. Molecular interactions, downstream pathway modulation, and pharmacokinetic exposure represent distinct levels of evidence. Cellular activity concentrations should not be equated directly with clinically relevant target engagement. Interpretation should consider unbound systemic and target-site exposure, tissue and intracellular distribution, metabolites, target accessibility, and formulation. Conversely, low circulating concentrations of the parent compound do not necessarily preclude local gastrointestinal, tissue-specific, metabolite-mediated, or microbiota-associated pharmacology.

**Table 2:** Pathway-Centred Evidence Map for Representative Phytochemicals.

Pathway/Node	Curcumin	Berberine	EGCG	Resveratrol	Principal Interpretation
NF-κB	Cellular/preclinical NF-κB suppression reported [42,43]; reduction in PBMC NF-κB was also evaluated as a human pharmacodynamic observation in a Phase II study [66]	NF-κB-associated modulation reported primarily at the preclinical/review level [7]; the experimentally supported complex I–AMPK mechanism is mechanistically distinct [44]	Cellular NF-κB modulation [21]; independently demonstrated 67LR binding should not be interpreted as evidence of direct NF-κB engagement [46]	NF-κB modulation is reported at the preclinical/review level [7]. SIRT1 can regulate NF-κB [61], but direct SIRT1 activation by resveratrol remains disputed [18]	Convergent NF-κB modulation does not imply a shared direct molecular target or common upstream mechanism
Nrf2/Keap1	Nrf2-associated cellular effects; electrophilic chemistry may contribute [7,41]	Nrf2-associated effects reported primarily at the preclinical/review level [7]	Nrf2-associated cellular/preclinical effects reported; redox/oxidation-dependent mechanisms may contribute, but direct Keap1 engagement remains unproven [7,16]	Nrf2-associated stress-response modulation reported primarily at the preclinical/review level [7]	Nrf2 activation can arise from oxidative, electrophilic, metabolic, or indirect signalling mechanisms and should not itself be interpreted as evidence of direct Keap1 engagement [38,39]
AMPK/mTOR/autophagy	Akt/mTOR- and autophagy-associated cellular/preclinical responses reported [42]	Functional inhibition of mitochondrial respiratory complex I with downstream AMPK activation is supported by experimental evidence [44]	AMPK/mTOR- and autophagy-associated effects are reported predominantly from cellular/preclinical studies [7,21]	PDE inhibition has been linked experimentally to indirect cAMP–Ca^2+^–CaMKKβ–AMPK signalling [47]	Convergence on AMPK/mTOR/autophagy does not establish direct AMPK engagement or a shared molecular mechanism. The AMPK–mTOR–ULK1 regulatory relationship is independently established [62]
STAT3	JAK/STAT3-associated cellular suppression [43]	JAK/STAT-associated suppression is reported at the preclinical/review [7]	Cellular STAT3 suppression [64]	Src–STAT3 suppression [65]	Predominantly cellular pathway evidence; molecular entry points differ
Pathway crosstalk/cooperativity	Network-level hypothesis; no inference of pharmacological synergy from pathway overlap alone	Network-level hypothesis; no inference of pharmacological synergy from pathway overlap alone	Network-level hypothesis; no inference of pharmacological synergy from pathway overlap alone	Network-level hypothesis; no inference of pharmacological synergy from pathway overlap alone	Crosstalk is not evidence of synergy; quantitative interaction modelling is required [27]

Evidence interpretation. The table distinguishes biochemical or biophysical target engagement, cellular pathway modulation, in vivo evidence, and human pharmacodynamic observations. Evidence of pathway modulation should not be interpreted as evidence that the phytochemical directly binds every component of that pathway. Similarly, convergence of several phytochemicals on NF-κB, Nrf2, AMPK/mTOR, or STAT3 does not by itself demonstrate common molecular targets, simultaneous multi-target engagement, pharmacological synergy, or therapeutic causality.

**Table 3:** Human Exposure–Activity Comparison for Representative Phytochemicals.

Compound	Representative Human Study Exposure	Measured Systemic Parent Exposure	Human Tissue/Local Exposure and Metabolites	Representative Mechanistic Activity or Affinity Context	Translational Interpretation
Curcumin	Approximately 570 mg of several formulations, with a conventional formulation also evaluated at 2280 mg [67]; 3.6 g/day for 7 days in colorectal cancer patients [48]	Unconjugated curcumin remained <2 nM under most formulation conditions; NovaSOL produced transient concentrations of approximately 6.7–38 nM [67]	After 3.6 g/day, parent curcumin reached 12.7 ± 5.7 nmol/g in normal colorectal tissue and 7.7 ± 1.8 nmol/g in malignant colorectal tissue; sulfate and glucuronide conjugates were also detected [48]	Many reported mechanistic cellular effects occur at micromolar concentrations [12,42]. Kroon et al. noted that even enhanced systemic exposure remained substantially below concentrations commonly used for many in vitro biological effects [67]	A substantial systemic exposure–activity mismatch exists for many mechanisms attributed to parent curcumin. However, measurable colorectal exposure can occur despite trace peripheral parent-compound exposure, indicating that local gastrointestinal pharmacology should be considered separately
Berberine	A single 400 mg oral dose was characterised in 20 healthy volunteers [49]. A more recent human PK study administered a single 1000 mg oral dose to 44 genotype-selected healthy participants [68]	After 400 mg, the mean parent-berberine Cmax was approximately 0.4 ng/mL (~1.2 nM) [14,49]. The 1000 mg study confirmed very low systemic exposure but revealed marked variability: approximately 29-fold variation in AUC and 32-fold variation in Cmax, with higher exposure in women than men [68]	Direct quantitative human tissue concentrations were not determined in the current core PK references. Parent berberine was measured in plasma, whereas phase I and phase II metabolites were characterised primarily in urine; CYP2D6 genotype and sex influenced aspects of metabolism and systemic exposure [68]	Experimental complex I/AMPK studies employ micromolar concentrations of berberine and demonstrate functional inhibition of mitochondrial respiratory complex I, with downstream AMPK activation [44]. These studies establish functional respiratory inhibition and downstream AMPK activation, but do not establish direct binding to a defined complex-I subunit	The nanomolar parent-plasma concentrations observed after conventional oral dosing are substantially below the micromolar concentrations used in many mechanistic experiments. Intestinal exposure, microbial transformation, tissue distribution and metabolites may therefore contribute to pharmacology, but direct human tissue exposure and target-engagement data remain insufficient to assign these mechanisms clinically
EGCG	Healthy volunteers received single doses of 200, 400, 600 or 800 mg EGCG [50]; In a separate tissue study, men with localised prostate cancer consumed six cups of green tea daily for 3–6 weeks before prostatectomy [51]	Mean EGCG Cmax increased from 73.7 ± 25.3 ng/mL at 200 mg to 438.5 ± 284.4 ng/mL at 800 mg [50]	After consumption of six cups of green tea daily before prostatectomy, EGCG and 4″-O-methyl-EGCG were detected in prostate tissue at 42.1 ± 32.4 pmol/g and 38.9 ± 19.5 pmol/g, respectively [51]	EGCG binding to 67LR has been reported with Kd ≈ 40 nM [46], whereas nuclear DNMT activity was competitively inhibited with K_i_ = 6.89 μM in a nuclear-extract assay [17]. Other cellular signalling effects are commonly reported at micromolar concentrations [21]	Exposure compatibility is mechanism-specific. The measured plasma EGCG concentrations are numerically compatible with the nanomolar 67LR affinity but remain below the 6.89 μM DNMT-inhibition constant. However, plasma Cmax cannot be equated with unbound receptor-site or intracellular nuclear exposure; therefore, neither receptor occupancy nor DNMT target engagement can be inferred from plasma concentration alone
Resveratrol	A human absorption study used a 25 mg oral dose [22]. Colorectal cancer patients received 0.5 or 1.0 g/day for 8 days before surgical resection [52]. A crossover PK study compared two 406 mg formulations in healthy fasting subjects [53]	After 25 mg, unchanged parent resveratrol remained <5 ng/mL (<~22 nM) despite ≥70% absorption, whereas circulating parent plus resveratrol-derived species reached approximately 2 μM [22]. After 406 mg, formulation had a major effect: unconjugated-parent Cmax was 1271.8 ± 496.4 ng/mL (~5.6 μM) with an HP-β-cyclodextrin formulation versus 44.1 ± 19.1 ng/mL (~0.19 μM) with the comparator formulation [53]	Parent resveratrol and multiple glucuronide and sulfate metabolites were detected in human colorectal tissue after 0.5–1.0 g/day [52]. Tissue exposure was highly heterogeneous: the highest mean parent concentrations in right-sided normal colorectal tissue were approximately 18.6 nmol/g after 0.5 g/day and 674 nmol/g after 1.0 g/day, while corresponding right-sided tumour values were approximately 8.33 and 94.1 nmol/g, respectively [52]	Resveratrol inhibited recombinant PDE1, PDE3 and PDE4 with IC50 values of approximately 6, 10 and 14 μM, respectively [47]. Direct SIRT1 activation with native substrates was not reproduced in an independent biochemical study and remains disputed [18]	Conventional low-dose parent exposure is far below the micromolar concentrations required for PDE inhibition. An exposure-enhancing formulation produced a parent Cmax that numerically approached the reported PDE1 IC_50_ of approximately 6 μM [47,53], demonstrating that formulation can materially alter exposure plausibility. This numerical overlap does not, however, demonstrate unbound intracellular PDE exposure, target occupancy, pharmacodynamic response or clinical efficacy. Local colorectal exposure after high oral doses likewise differs substantially from peripheral exposure and should be interpreted separately

**Table 4:** Clinical Evidence and Translational Interpretation of Representative Phytochemical Interventions.

Intervention	Clinical Setting and Design	Sample Size/Intervention	Major Endpoint or Observation	Human PK/PD or Mechanistic Information	Translational Interpretation
Curcumin [66]	Nonrandomized, open-label Phase II trial; advanced pancreatic cancer	25 enrolled; 21 evaluable for response; 8 g/day oral curcuminoid formulation	Clinical biological activity was observed in a small number of patients; objective anticancer efficacy was not established	Circulating curcumin-derived species were detected at low concentrations, predominantly as conjugates. PBMC NF-κB and COX-2 expression and circulating cytokines were evaluated as pharmacodynamic/correlative measures	Demonstrates tolerability and limited human biological activity, but does not establish clinically validated anticancer efficacy, exposure-compatible multi-target engagement, or causal pathway mediation
Curcumin [75]	Nonrandomized, open-label Phase IIa trial; colorectal neoplasia	44 participants began treatment; 41 completed; 2 or 4 g/day for 30 days	ACF number decreased significantly in the 4 g/day group, whereas PGE_2_, 5-HETE, and K_i_-67 were not significantly altered	Serum and rectal mucosal curcumin concentrations were assessed; plasma curcumin/conjugate exposure increased in the 4 g/day group.	The reduction in ACF represents an intermediate finding rather than a validated surrogate for colorectal cancer prevention and does not establish a specific molecular mechanism
Green-tea extract [76]	Randomised, double-blind, placebo-controlled Phase II trial; high-risk oral premalignant lesions	41 enrolled; 39 evaluable; GTE 500, 750, or 1000 mg/m^2^ three times daily versus placebo for 12 weeks	Overall clinical lesion response was numerically higher with GTE than placebo, but was not statistically significant; higher-dose groups showed a significant dose–response trend, whereas histological improvement was not significant	Serial lesion biopsies were used for biomarker assessment; plasma EGCG and caffeine concentrations were also evaluated	Provides a short-term lesion-response signal but does not establish cancer-preventive efficacy, purified EGCG efficacy, or EGCG-specific molecular target engagement
Berberine [57]	Randomised, double-blind, placebo-controlled multicentre trial; newly diagnosed type 2 diabetes	409 randomised; berberine alone, probiotics plus berberine, probiotics alone, or placebo for 12 weeks	HbA1c reduction was significantly greater in the berberine-containing groups than in the placebo or probiotics-alone groups	Metagenomic and metabolomic analyses implicated altered microbial bile-acid metabolism, including *Ruminococcus bromii*-associated deoxycholic-acid biotransformation	Demonstrates improvement in the glycaemic endpoint HbA1c and supports a microbiome-associated mechanistic contribution, but does not establish that this mechanism accounts for the full metabolic response or demonstrate network-level polypharmacology
Curcumin [78]	Randomised, double-blind, placebo-controlled trial; mild-to-moderate Alzheimer’s disease	36 participants received placebo, 2 g/day, or 4 g/day for 24 weeks	No significant benefit was demonstrated for cognitive outcomes or measured Alzheimer-related plasma/CSF biomarkers	Native curcumin exposure in plasma was low, while conjugated metabolites predominated; native curcumin was not detected in CSF	Does not provide convincing evidence of clinical or biomarker efficacy under the studied conditions and illustrates the difficulty of achieving pharmacologically relevant CNS exposure
Resveratrol [69]	Randomised, double-blind, placebo-controlled multicentre Phase II trial; mild-to-moderate Alzheimer’s disease	119 randomised; 52 weeks; resveratrol initiated at 500 mg once daily and escalated every 13 weeks to 1 g twice daily	Selected biomarker trajectories were altered; no consistent benefit occurred on major cognitive or global measures, although the decline in ADCS-ADL was smaller in the resveratrol group	Parent resveratrol and major metabolites were measurable in plasma and CSF; detailed pharmacokinetic analyses were performed in a subset	Demonstrates CNS exposure of resveratrol-derived molecular species and selected pharmacodynamic activity, but not definitive clinical efficacy or specific neural target engagement

**Table 5:** Translational Framework Integrating Prediction, Validation, and Clinical Translation.

Stage	Current Approach	Principal Limitation	Recommended Improvement	Evidence Position
Phytochemical characterisation	Chemical and phytochemical databases	Identity, purity, composition, and batch variability	Validated chemical identity, composition, purity, and stability	Foundation for subsequent evidence
Target prediction	Network pharmacology, docking, and AI-assisted prediction	False positives, database dependence, structural assumptions	Independent prediction methods, exposure filtering, and prospective validation	T0: hypothesis generation
Biochemical validation	Binding or activity assays	Assay interference and non-physiological conditions	Orthogonal biochemical/biophysical assays and concentration–response analysis	T1: biochemical/molecular evidence
Cellular validation	Reporter, phosphorylation, transcriptional, and phenotypic assays	Downstream responses may not identify the direct target	Use proximal pathway readouts and mechanistically informative cellular assays	T2: cellular/pathway evidence
Functional/genetic validation	Genetic perturbation and target-engagement approaches	Genetic dependency may be indirect; engagement alone does not establish causal contribution	Combine biochemical evidence, CETSA or other engagement methods, genetic perturbation, rescue, or resistant mutants	T3: functional/causal and/or target-engagement evidence; these are complementary forms of evidence
Pharmacokinetic assessment	Plasma and systemic PK	Plasma concentration may not represent target-site exposure	Tissue and metabolite measurements and validated PBPK modelling	Exposure constraint across evidence tiers
Drug delivery	Conventional and advanced formulations	Increased exposure does not establish target engagement or efficacy	Evaluate formulation → PK → tissue exposure → target engagement → PD → clinical outcome	Translational enabling layer
Network/pathway analysis	Network topology and pathway enrichment	Convergence may be mistaken for causality	Functional perturbation and orthogonal molecular validation	Primarily T0–T2
Single-cell/spatial profiling	scRNA-seq and spatial omics	Molecular responses are predominantly associative	Cell-type-resolved validation and functional perturbation	Hypothesis-refinement layer
AI-assisted prediction	GNNs, multimodal models, and knowledge graphs	Dataset bias, applicability limits, and validation uncertainty	Independent validation and prospective experimental confirmation	Primarily T0
Clinical translation	Clinical and biomarker studies	Exposure mismatch and uncertainty regarding mechanistic attribution	Exposure-informed studies with validated PD measures and clinically meaningful endpoints	T5: human evidence; interpretation depends on endpoint

## Data Availability

No new data were created or analyzed in this study. Data sharing is not applicable to this article.
